# Structure of polyhydroxyalkanoate (PHA) synthase PhaC from *Chromobacterium* sp. USM2, producing biodegradable plastics

**DOI:** 10.1038/s41598-017-05509-4

**Published:** 2017-07-13

**Authors:** Min Fey Chek, Sun-Yong Kim, Tomoyuki Mori, Hasni Arsad, Mohammed Razip Samian, Kumar Sudesh, Toshio Hakoshima

**Affiliations:** 1 Structural Biology Laboratory, Nara Institute of Science and Technology, 8916-5 Takayama, Ikoma, Nara 630-0192 Japan; 2 Advanced Medical and Dental Institute, Universiti Sains Malaysia, Bertam 13200, Kepala Batas, Penang Malaysia; 3 School of Biological Sciences, Universiti Sains Malaysia, 11800 Penang, Malaysia

## Abstract

Polyhydroxyalkanoate (PHA) is a promising candidate for use as an alternative bioplastic to replace petroleum-based plastics. Our understanding of PHA synthase PhaC is poor due to the paucity of available three-dimensional structural information. Here we present a high-resolution crystal structure of the catalytic domain of PhaC from *Chromobacterium* sp. USM2, PhaC_*Cs*_-CAT. The structure shows that PhaC_*Cs*_-CAT forms an α/β hydrolase fold comprising α/β core and CAP subdomains. The active site containing Cys291, Asp447 and His477 is located at the bottom of the cavity, which is filled with water molecules and is covered by the partly disordered CAP subdomain. We designated our structure as the closed form, which is distinct from the recently reported catalytic domain from *Cupriavidus necator* (PhaC_*Cn*_-CAT). Structural comparison showed PhaC_*Cn*_-CAT adopting a partially open form maintaining a narrow substrate access channel to the active site, but no product egress. PhaC_*Cs*_-CAT forms a face-to-face dimer mediated by the CAP subdomains. This arrangement of the dimer is also distinct from that of the PhaC_*Cn*_-CAT dimer. These findings suggest that the CAP subdomain should undergo a conformational change during catalytic activity that involves rearrangement of the dimer to facilitate substrate entry and product formation and egress from the active site.

## Introduction

Biodegradable plastics (bioplastics) have attracted much interest in the field of biotechnology with expectations of a potential contribution to reducing solid waste, such as microplastics in oceans and carbon dioxide emissions from petroleum-based plastics, since bioplastics degrade without leaving polluting substances. A prototypical bioplastic is polyhydroxyalkanoate (PHA), which could replace conventional plastics since it possesses comparable thermal stability and various physical attributes such as elastomeric properties^1–3^. PHAs are a class of biodegradable polyesters synthesized by a wide range of bacteria and halophilic archaea, and can be produced from renewable resources such as plant biomass, oils, sugars, and even carbon dioxide^4–7^. When microorganisms are exposed to excess carbon sources and limited amounts of other nutrients in the surrounding environment, PHAs are synthesized as water-insoluble inclusions, which are stored as carbon reserves and degraded by PHA depolymerase (PhaZ) during carbon starvation^8, 9^. At present, PHAs have been investigated for potential incorporation and utilization in industries producing packaging or cosmetics, agriculture, pharmacology, and medicine^10–12^.

**Figure 1:**
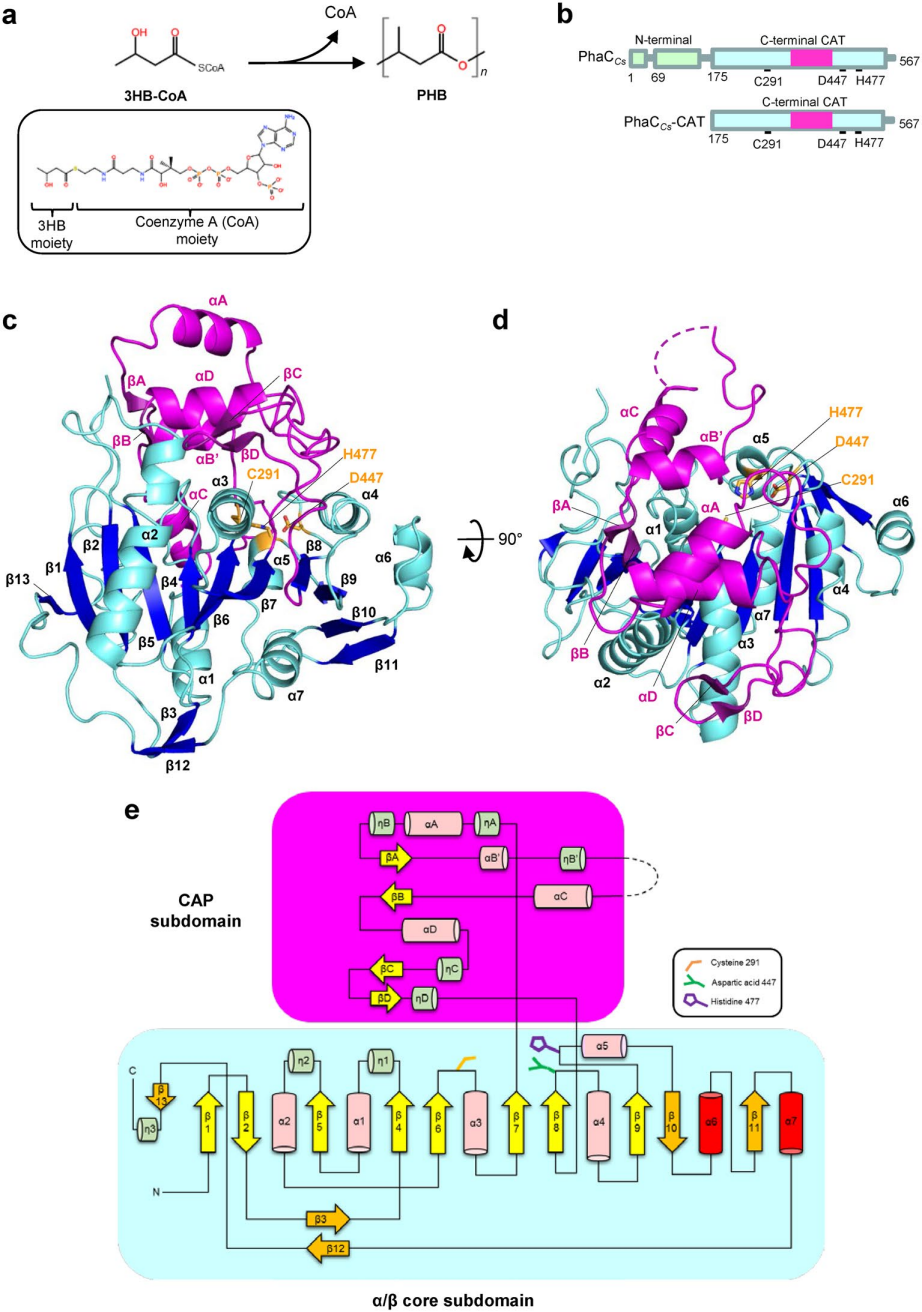
Structure of PhaC_*Cs*_-CAT. (**a**) Polymerization reaction catalyzed by PhaC. Polymerization of the substrate, 3-hydroxybutyryl coenzyme A (3HB-CoA), into poly 3-hydroxylbutyrate (PHB) is catalyzed by PhaC with release of CoA. The chemical structure of 3HB-CoA is shown in the inset. (**b**) Domain organization of PhaC_*Cs*_. PhaC_*Cs*_ consists of two domains, the N-terminal domain which is important for stabilizing dimeric PhaC and the C-terminal catalytic (CAT) domain containing conserved active site including triad residues Cys, His and Asp. (**c**) A side view of PhaC_*Cs*_-CAT. The catalytic domain of PhaC_*Cs*_ comprises the α/β core subdomain (residues 175–318, 439–562 in cyan) and the CAP subdomain (residues 319–438 in violet). The side chains of the catalytic triad (Cys291, His477 and Asp447) are shown in orange. (**d**) As in b, but a top view of PhaC_*Cs*_-CAT. The active site is covered by the CAP subdomain. (**e**) Schematic presentation of the secondary structure topology of PhaC_*Cs*_-CAT (mol B). The CAT domain contains the CAP and core subdomains. The core subdomain comprises 13 strands and 10 helices. Nucleophilic Cys291 is located between β6 and α3, conserved His477 is located between β9 and α5, and Asp447 is located between β8 and α4. The CAP subdomain is connected from β7 and back to the core domain through β8. The catalytic triad is covered by the CAP subdomain which blocks the substrate entry pathway. The other strands (orange) and helices (red) shown represent additional secondary structures observed when comparing the canonical α/β hydrolase fold.

PHA synthase (PhaC) is the key enzyme involved in PHA biosynthesis and functions by polymerizing monomeric hydroxyalkanoate substrates^3, 13, 14^. At present, a total of 14 pathways have been reported which lead to PHA synthesis^15^. PHA synthases have been categorized into four major classes based on their primary sequences, substrate specificity, and subunit composition^3, 16^. Class I comprises enzymes consisting of only one type of PhaC, which forms a homodimer, while Class II contains two types of synthases, PhaC1 and PhaC2. Class III and IV synthases form heterodimers, comprising PhaC-PhaE and PhaC-PhaR, respectively. Class I, III and IV synthases tend to favor short-chain-length (SCL) monomers comprising C3-C5 carbon chain lengths. A typical example of a C4 SCL monomer is (*R*)-3-hydroxybutyrate (3HB), and PhaC polymerizes the acyl moieties of 3-hydroxybutyryl-coenzyme A (3HB-CoA) to the high molecular weight PHA product poly-hydroxybutyrate (PHB) (Fig. 1a). Class II synthases favor medium-chain-length (MCL) monomers comprising C6-C14 carbon chain lengths, such as the C6 monomer 3-hydroxyhexanoate (3HHx).

Since PHAs comprising different monomeric units such as mixtures of SCL and MCL hydroxyalkanoates possess better thermal and physical properties compared with homo-polymers, efforts have been taken to improve the performance of these synthases by broadening their substrate specificity through enzyme engineering^17, 18^. Alternatively, the isolation and screening of natural synthases with higher performances and broader substrate specificity is still a common practice. A Class I PHA synthase gene (*phaC*_*Cs*_) from a newly isolated bacteria, *Chromobacterium* sp. USM2, encodes an enzyme that produces poly(3-hydroxybutyrate-*co*-3-hydroxyhexanoate) [P(3HB-*co*-3HHx)] copolymer and poly(3-hydroxybutyrate-*co*-3-hydroxyvalerate-*co*-3-hydroxyhexanoate) [P(3HB-*co*-3HV-*co*-3HHx)] terpolymer, which are ideal materials for use in industrial products due to their softness and flexibility^17, 19, 20^. Moreover, an *E. coli* transformant harboring *phaC*_*Cs*_ was shown to accumulate poly(3-hydroxybutyrate) [P(3HB)] up to 76 weight percent within 24 hours of cultivation. Furthermore, Strep2-tagged PhaC_*Cs*_ showed a specific activity of 238 U/mg, which is almost 5-fold higher than the activity of PhaC from *Cupriavidus necator*/*Ralstonia eutropha* (PhaC_*Cn*_), a commonly used PHA synthases in previous studies^21^.

The high performance and ability of PhaC_*Cs*_ to utilize and incorporate both SCL and MCL monomers has garnered great attention from research communities. The hitherto lack of a three-dimensional structure of PhaC has limited our understanding of the polymerization mechanisms involved, the factors that determine chain length and polydispersity, and the molecular basis of the enzyme specificity. An understanding of the mechanisms based on three-dimensional structures should play an important role in determining the feasibility of these materials for industrial use. In this study, the first three-dimensional structure of the catalytic domain of PhaC_*Cs*_ (PhaC_*Cs*_-CAT) was determined at 1.48 Å resolution by X-ray crystallography. The overall structure of the α/β hydrolase fold and the active site geometry is compared with the structure of a related enzyme, gastric lipase. In the course of our structural studies, two independent structural investigations of the catalytic domain of PhaC from *Cupriavidus necator* (the old code is *Ralstonia eutropha*), PhaC_*Cn*_-CAT (both at 1.8 Å), have been reported^22, 23^. Although these studies have essentially reported the same structure comprising a partially open form, the proposed enzyme mechanisms differ. Moreover, our determined structure appears to differ from these structures with respect to the dimer arrangement and state of the enzyme. Our structure comprises a closed form, which has no visible path for substrate access to the active site located inside of the enzyme. Our three-dimensional structure of PhaC_*Cs*_ together with PhaC_*Cn*_ provides valuable information concerning the enzyme dynamics and should assist in delineation of the enzymatic mechanisms utilized by the important and related synthases, in addition to providing the basis for facilitating structure-based improvement of the enzymatic activity.

## Results

### Protein preparation and structural determination

Database searches showed that PhaC members lack overall amino-acid sequence similarity with any enzymes whose three-dimensional structures have been determined. However, the C-terminal region of the enzymes is conserved among PhaC’s and possesses a lipase box sequence, Gx_1_Sx_2_G (where x_1_ and x_2_ represent any amino acid residue, and Ser is replaced with Cys in PhaC), which suggests that PhaC may belong to the lipase superfamily^24–26^ and that this C-terminal region is the catalytic domain (Fig. 1b). We observed that full-length PhaC_*Cs*_ possesses a protease-sensitive region between the N-terminal region and the C-terminal catalytic domain. This region seems to be conformationally flexible and therefore prevent the full-length protein from crystallization. In contrast, the catalytic domain is stable and produced well-diffracted crystals. The structure of the catalytic domain (residues 175–567), hereafter referred to as PhaC_*Cs*_-CAT, was determined by a single-wavelength anomalous dispersion (SAD) method and refined to 1.48 Å resolution. Crystal and structure determination data are summarized in Supplementary Table 1. PhaC is generally depicted to form a dimer which is an important form for its functional activity^27–29^. Consistently, our crystal contains two PhaC_*Cs*_-CAT molecules in the asymmetric unit and these molecules are found to form a dimer as described below.

### Overall structure of the catalytic domain of PhaC_*Cs*_

PhaC_*Cs*_-CAT is folded into a globular structure which belongs to the α/β hydrolase super family and comprises α/β core and CAP (hereafter referred to as CAP) subdomains (Fig. 1c–e). The core subdomain forms 13 β-strands (β1-β13) and comprises the central eleven-stranded β-sheet, which contains 9 parallel and 2 antiparallel β-strands with strand connectivity β13-β1-β2-β5-β4-β6-β7-β8-β9-β10-β11, and seven α-helices (α1-α7) located at both sides of the central β-sheet: with α1, α5 and α7-helices on one side, and α2, α3, α4 and α6 on the other side (Fig. 1e). In addition to these α-helices and β-strands, eight short 3_10_-helices (η1-η3 in the core and ηA-ηD in the CAP subdomains) are found. Besides the central β-sheet, the core subdomain forms an additional two-stranded antiparallel β-sheet (β3 and β12) which is located beneath the core subdomain (Fig. 1c,e). As seen in the α/β hydrolase fold, the central β-sheet of PhaC_*Cs*_-CAT displays a left-handed super-helical twist with the first β1 strand crossing the eighth β-strand (β9) at ~90° and the last strand (β11) at ~180°. In the core subdomain, Class III and IV synthases lack most of the C-terminal additional region following β10 strand (Supplementary Fig. 1).

The CAP subdomain is formed by 120 residues (residues 319–438), which is topologically projected from the regular α/β fold (residues 186–318 and residues 439–562) by protruding from β7 strand and connecting back to β8 strand (Fig. 1c). The CAP subdomain comprises 4 α-helices (αA, αB’, αC and αD) and four β-strands (βA-βD), and encompasses an 11-residue missing segment (373-YVVNNYLLGKT-383 in chain B) located at the random coil between ηB’ and αC helices. In comparison with PhaC_*Cn*_-CAT, αB’ and ηB’ helices are found to be metastable and undergo a conformational transition to one helix in an open form (see below). The CAP subdomain covers a cleft containing the active site residues that comprise the catalytic triad (Fig. 1d).

PHA synthases have previously been described by comparison with α/β hydrolase of the lipase family^24^. The current structure of PhaC_*Cs*_-CAT, however, reveals several unique structural characteristics, which are absent in the canonical α/β hydrolase fold of lipases constructed by the α/β core comprising eight β-strands and six α*-*helices. Structural comparison with mammalian lipases^30, 31^ showed differences in their architecture (Supplementary Fig. 2). The major difference in the core subdomains is due to the additional presence of five β-strands (β3, β10, β11, β12, and β13) and two α*-*helices (α6 and α7) in PhaC_*Cs*_-CAT. An additional major deviation is found in the helical packing of the CAP subdomain: the N-terminal 3_10_-helix (ηA) and the C-terminal two helices (αC and αD) adopt a comparable orientation and position with that of the corresponding helices of lipases, while the other helices show no similarity due to conformational rearrangement.

#### Catalytic triad

**Figure 2:**
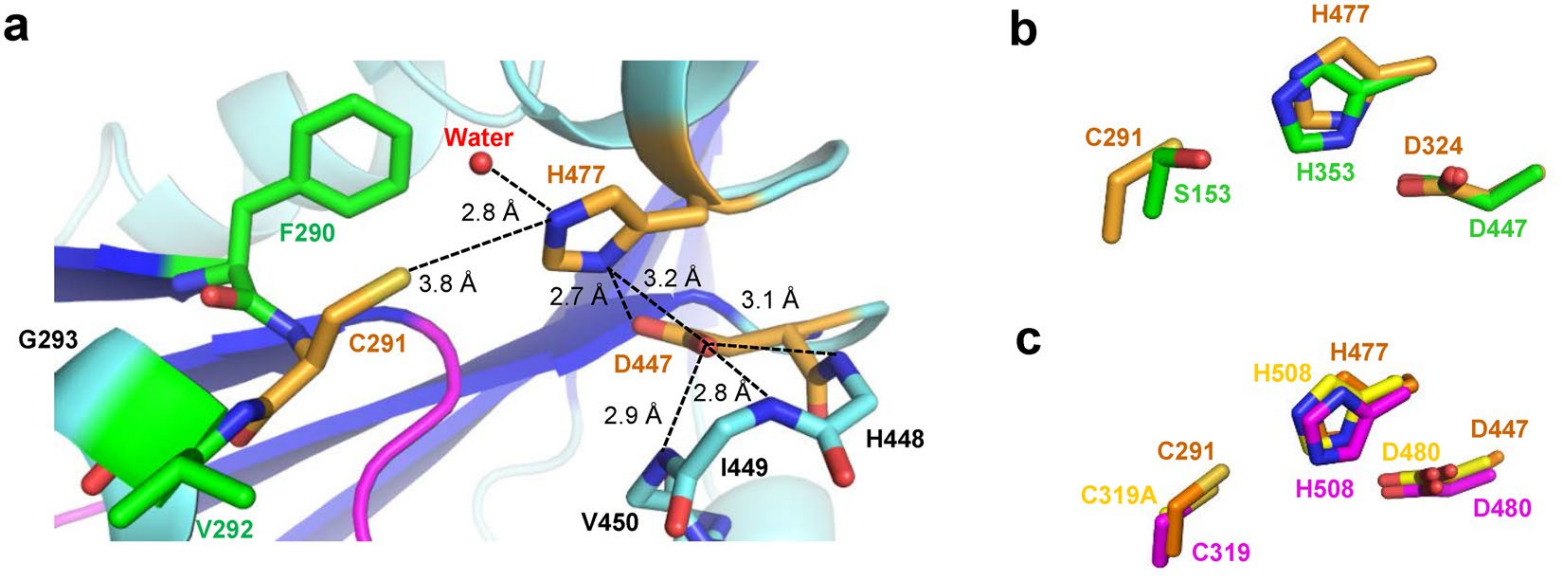
The catalytic residues of PhaC_*Cs*_-CAT. (**a**) A close-up view of the catalytic site of PhaC_*Cs*_-CAT. The catalytic triad residues (orange) comprise Cys291, His477 and Asp447, with a hydrogen bond between His477 and Asp447. The catalytic center Cys291 is located at the nucleophilic elbow sandwiched with nonpolar residues Phe290 and Val292 (green). Hydrogen bonds are indicated by broken lines. (**b**) Overlay of the catalytic triad of PhaC_*Cs*_-CAT (orange) onto human gastric lipase (1HLG in green). (**c**) Overlay of the active site with the catalytic triad of PhaC_*Cs*_-CAT (orange) onto those of PhaC_*Cn*_-CAT (5T6O in yellow; 5HZ2 in magenta). The imidazole ring of the His residue of PhaC_*Cn*_-CAT (magenta) is flipped from the other two.

The active site residue Cys291 is located at the turn between β6 strand and α3 helix. This turn contains a conserved lipase box-like motif Gx_1_Cx_2_G (where x_1_ is Phe290 and x_2_ is Val292 in PhaC_*Cs*_) and forms a “nucleophilic elbow” found in α/β hydrolases, holding the catalytic cysteine residue at the top position of the elbow (Fig. 2a). Cys291 forms the catalytic triad with His477 (located at the N-end of α5 helix) and Asp447 (located at the loop between β8 strand and α4 helix, β8-α4 loop). All of these catalytic residues are essential for the production of P(3HB) (Supplementary Fig. 3). The main-chain amide group of Val292 is found to have an orientation suitable for forming the oxyanion hole. The catalytic triad is located at the N-terminal edge of the central parallel β-sheet. Five α-helices (α1-α5) point the N-terminal edge with their N-terminal ends in a manner so that their electric dipoles stabilize binding to the negatively charged molecule, presumably alkyl-CoA. In our crystal structure, the active site is covered by the CAP subdomain, and no path was found from the protein surface which would allow substrate 3HB-CoA to enter the active site (Fig. 1d).

The side chain conformation of Asp447 is stabilized by formation of three hydrogen bonds with the main chain NH groups of β8-α4 loop residues His448, Ile449 and Val450 (3.1 Å, 2.8 Å and 2.9 Å, respectively). The Asp447 side chain forms another hydrogen bond (2.7 Å) with His477 (Nδ), which forms a hydrogen bond to a water molecule inside the active site pocket (Fig. 2a). One of the ring nitrogen atoms (Nε) is close (3.8 Å) to the sulfur atom of Cys291. The relative position of the three catalytic residues corresponds well with that of lipases (Fig. 2b), suggesting a similar mechanism by which these three residues catalyze the esterification reaction. In PHA synthesis catalyzed by PhaC, active center Cys291 engages in nucleophilic attack of the thioester of acyl-CoA (e.g. 3HB-CoA), which becomes acylated. This attack is accelerated by deprotonation of the thiol group of Cys291 by His477. In our structure, Asp447 assists His477, probably with respect to deprotonation of Cys291, but also in terms of acting as a general base catalyst by accelerating deprotonation of the 3-hydroxyl group of HB-CoA^24, 32^.

#### Clusters of water molecules at the active site

**Figure 3:**
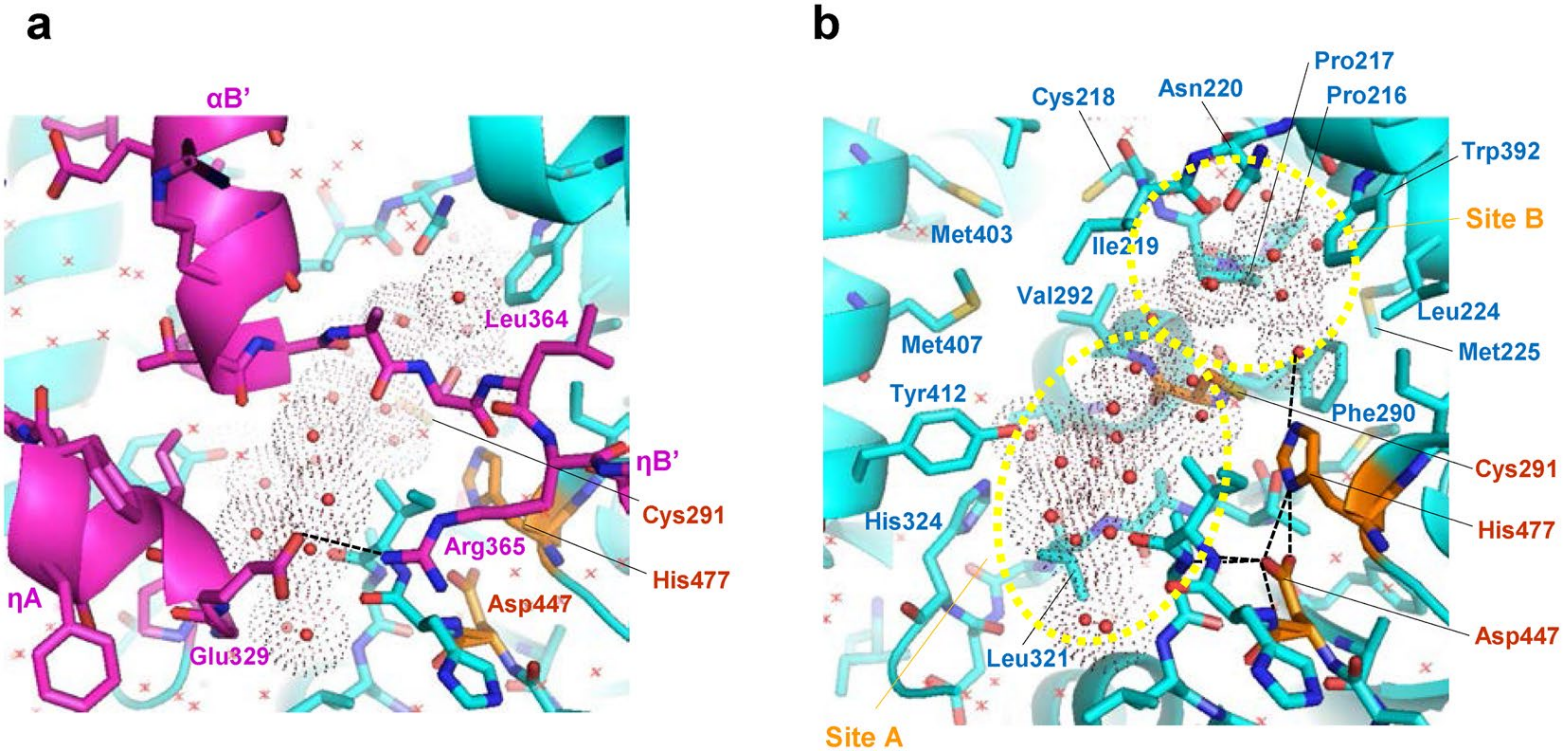
Water molecules at both sides of the nucleophilic elbow of PhaC_*Cs*_-CAT. (**a**) A cluster of water molecules (red balls with dotted surfaces) is located at the active site cavity around the nucleophilic elbow. The cavity is covered by the LID region of the CAP subdomain (magenta). Other water molecules are shown as red crosses. Color codes are the same as in Fig. 2. The hydrogen bond between Glu329 and Arg365 is indicated by a broken line. (**b**) As in **a**, but without the LID region. Hydrogen bonds involving the catalytic residues are shown as broken lines. Water molecules are divided into two groups, one group at Site A and the other at Site B. The cavity is mostly hydrophobic although Tyr412 and His324 are located at Site A and Asn220 at Site B. Hydrogen bonds are indicated by broken lines.

Our high resolution (1.48 Å) structure clearly elucidated the presence of water molecules inside and outside of the enzyme. We found several clusters of water molecules at the interface between the CAP and α/β core subdomains, suggesting potential mobility of the CAP subdomain, which may undergo a dynamic structural change during the catalytic action of the enzyme. Interestingly, active center Cys291 is located at the cavity filled with a cluster of water molecules, which is directly covered by part (Pro327-Pro386) of the CAP subdomain (Fig. 3a). This segment contains ηA, αA, ηB, αB’ and ηB’ helices and corresponds to the segment Thr355-Pro419 of PhaC_*Cn*_. The cluster of water molecules could be divided into two groups by the nucleophilic elbow (Fig. 3b). One group resides in a part of the cavity (Site A) formed by Val292 from the nucleophilic elbow and Val295 from α3 helix with two polar residues, Tyr412 from αD helix and His324 from β7-ηA loop, and other nonpolar residues (Leu321 from β7-ηA loop, Ile449 and Val450 from β8-α4 loop, Met407 from αD helix and Ile219 from β4-η1 loop). The other group resides in the nonpolar cavity (Site B) formed by Phe290 from the nucleophilic elbow with other nonpolar residues (Pro216, Pro217, Ile219 from β4-η1 loop, Leu224 and Met225 from η1 helix, and Trp392 from αC helix). Asn220 is the only polar residue that faces this water cluster.

#### Conformational changes in the CAP subdomain

**Figure 4:**
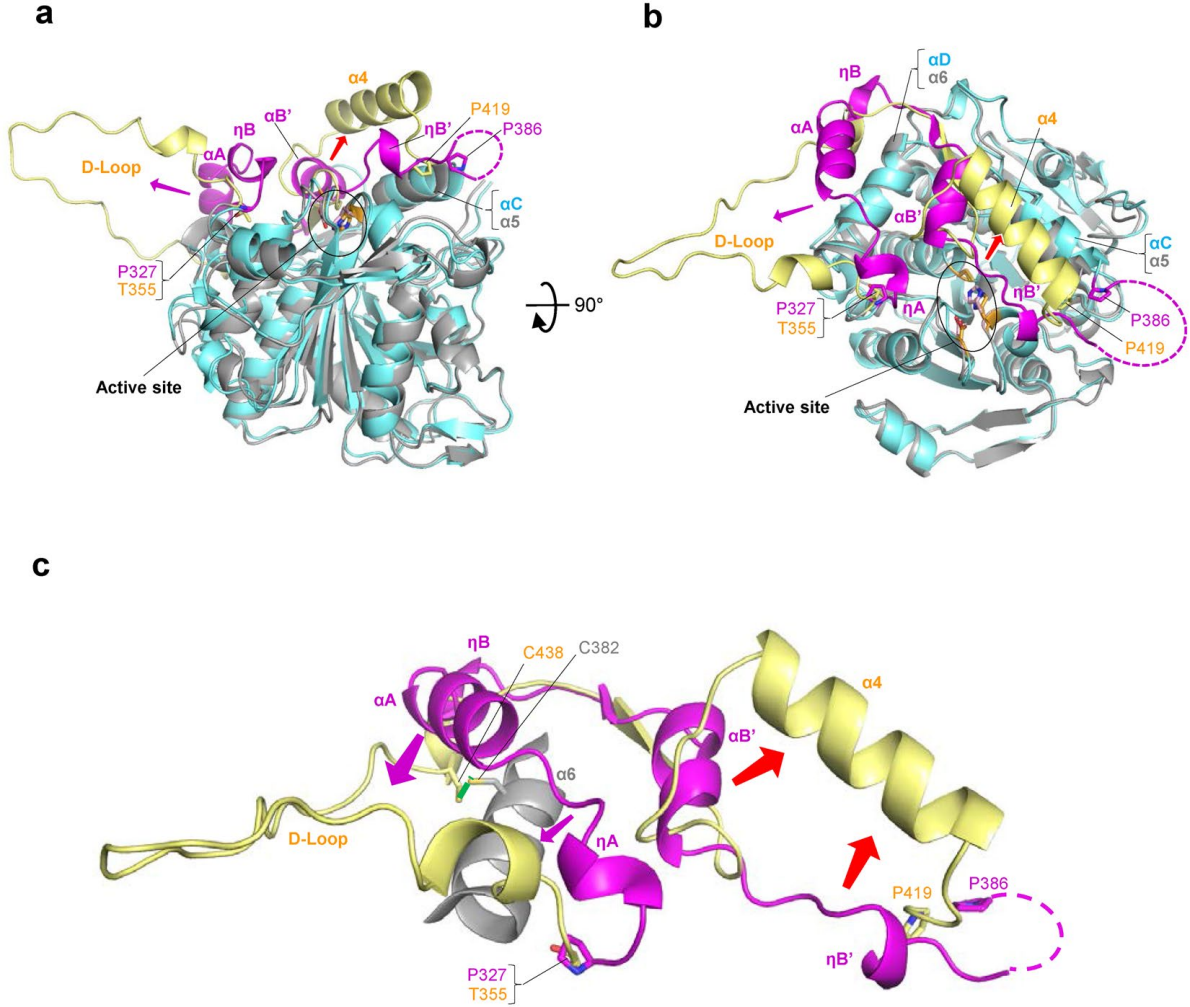
Conformational changes in the CAP subdomain as revealed by structural comparison of PhaC_*Cs*_-CAT and PhaC_*Cn*_-CAT structures. (**a**) A side-view of the overlay between PhaC_*Cs*_-CAT (cyan and magenta) and PhaC_*Cn*_-CAT (gray and yellow) with an overall r.m.s. deviation of 2.7 Å for Cα carbon atoms. The major conformational deviations are observed in part of the CAP subdomain, the LID region (residues Pro327–Pro386 in magenta) of PhaC_*Cs*_-CAT, which corresponds to the segment Thr355–Pro419 (yellow) of PhaC_*Cn*_-CAT. Arrows indicate conformational transitions of the LID region from PhaC_*Cs*_-CAT to PhaC_*Cn*_-CAT. (**b**) As in **a**, but a top-view down to the active site. A narrow path to the active site was found in PhaC_*Cn*_-CAT, whereas the path is covered by the LID region in PhaC_*Cs*_-CAT. (**c**) To clarify the conformational transitions found by the structural comparison of PhaC_*Cs*_-CAT and PhaC_*Cn*_-CAT, overlay of the LID regions of the two structures are shown. Unfolding of ηA, αA and ηB helices of PhaC_*Cs*_-CAT into a long flexible D-loop found in PhaC_*Cn*_-CAT is indicated by magenta arrows, and refolding of αB’ and ηB’ helices and their linker loop of PhaC_*Cs*_-CAT into a long α4 helix in PhaC_*Cn*_-CAT by red arrows. The disulfide bond between Cys328 and Cys438 of PhaC_*Cn*_-CAT is shown in green.

PhaC_*Cs*_ displays 46% amino acid sequence identity with PhaC_*Cn*_, which also belong to the Class I group of PHA synthases. It is interesting and instructive for our understanding of this enzyme to compare the structure of our PhaC_*Cs*_-CAT with PhaC_*Cn*_-CAT^22, 23^. As expected, the α/β core subdomains resemble each other and the relative positions of the Cys-His-Asp residues of the catalytic triads are well conserved, whereas the imidazole ring of the His residue is flipped in the structure determined by the KNU group^23^ (Fig. 2c). Our structural comparison reveals unexpected structural deviations in the CAP subdomain (Fig. 4). Prominent conformational deviations are observed in part of the CAP subdomain, the LID region (residues Pro327–Pro386 in magenta) of PhaC_*Cs*_-CAT, which corresponds to the segment Thr355–Pro419 of PhaC_*Cn*_-CAT. These deviations could be interpreted by two dynamic conformational transitions (Fig. 4c). One involves an unfolding of ηA, αA and ηB helices of PhaC_*Cs*_-CAT into a long flexible D-loop found in PhaC_*Cn*_-CAT. The other involves a folding of αB’ and ηB’ helices and their linker loop of PhaC_*Cs*_-CAT into the long α4 helix in PhaC_*Cn*_-CAT with a large shift in positions that uncovers a path to the active site. Interestingly, the segment (Leu402–Asn415) forming α4 helix of PhaC_*Cn*_-CAT has a conserved sequence among Class I and II PHA synthases (Supplementary Fig. 1, green box), whereas the corresponding segment (Leu369–Lys382) of PhaC_*Cs*_-CAT displays a disordered structure. This difference could be caused in part by the difference in dimerization modes as discussed below.

#### An artificial disulfide bond formation may induce the conformational change in the CAP subdomain of PhaC_*Cn*_-CAT

We have examined the structural details in an effort to arrive at a possible mechanism by which the conformational difference between PhaC_*Cs*_-CAT and PhaC_*Cn*_-CAT is induced at the CAP subdomains, and found that one disulfide bond is formed between Cys382 and Cys438 in the PhaC_*Cn*_-CAT structures^22, 23^ (Fig. 4c). These Cys residues are non-conserved and no such disulfide bond can be formed in our PhaC_*Cs*_-CAT structure. In the PhaC_*Cn*_-CAT structures, Cys382 is in the insertion segment specific to PhaC_*Cn*_ and located in part of the D loop of the LID region, which displays a distinct conformation from our PhaC_*Cs*_-CAT structure. In contrast, Cys438 is located at a common helix corresponding to αD helix of our PhaC_*Cs*_-CAT structure. It is likely that disulfide bond formation induces the long D-loop and stabilizes the partially open conformation of the CAP subdomain, which is distinct from the closed conformation of our PhaC_*Cs*_-CAT structure. It should be noted that PhaCs are cytoplasmic enzymes that act under reduced conditions of the redox potential, indicating that artificial formation of the disulfide bond may have occurred during crystallization. However, it may be possible that artificial disulfide bond formation contributes in trapping the partially open state of the CAP subdomain in an open-closed equilibrium in solution.

#### Dimeric structure of PhaC_*Cs*_-CAT

PHA synthases are known to exist in an equilibrium between monomer and dimer in solution and are believed to possess full catalytic activity in the dimeric form^33–36^. In an effort to analyze the equilibrium of PhaC_*Cs*_ in the absence or presence of substrate (DL-3HB-CoA), analytical ultracentrifugation (AUC) was employed utilizing a sedimentation velocity method (Supplementary Fig. 4a). The analysis revealed that at low concentration (5 µM), free PhaC_*Cs*_ (full-length, 63.4 kDa) exists in an equilibrium between monomeric and dimeric forms, with the monomeric form being dominant at this concentration. In the presence of substrate at a 10-fold molar ratio (50 µM), the equilibrium is shifted to include a higher order oligomer (presumably a tetramer). The monomer-dimer equilibrium was further analyzed by size exclusion chromatography (SEC) at a concentration of 30 µM with full-length PhaC_*Cs*_ and PhaC_*Cs*_-CAT (Supplementary Fig. 4b). In the absence or presence of substrate (500 µM), full-length PhaC_*Cs*_ exists in a monomeric-dimeric equilibrium with the monomeric form being dominant, which is similar to the results from analytical ultracentrifugation. Compared with this result, the monomeric form of PhaC_*Cs*_-CAT is more dominant and only a trace amount of the dimeric form was detected in SEC. Further analyses of the monomeric-dimeric equilibrium by AUC showed that the dimeric form of PhaC_*Cs*_-CAT exists and is stabilized at a high protein concentration (20 µM) (Supplementary Fig. 4c). In SEC, monomers are separated from each other in the resin and may fail to re-associated to form dimers again. Thus, the monomeric form seems to be apparently stabilized in SEC.

**Figure 5:**
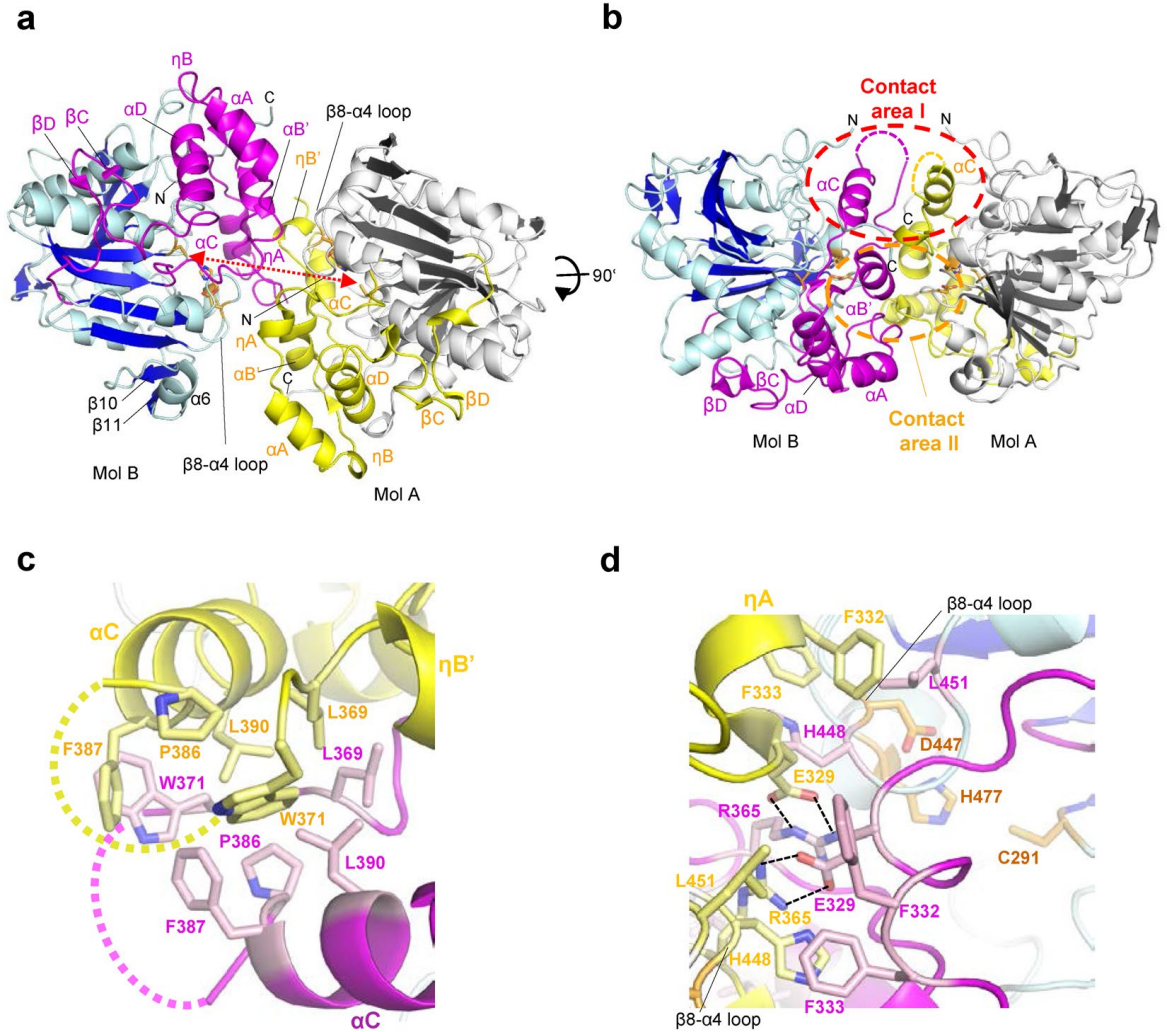
PhaC_*Cs*_-CAT forms a dimer in the crystal. (**a**) A top-view of the PhaC_*Cs*_-CAT dimer along the pseudo-dyad axis. The color codes of mol B are the same as in Fig. 1, with mol A core (gray) and CAP (yellow) subdomains shown. The distance between the catalytic cysteine residues is 28.1 Å (red double-headed arrow). (**b**) As in **a**, but the side-view. Contact areas I (red) and II (orange) are indicated by broken circles. The N-termini of both protomers are located at the same side of the dimer. (**c**) Contact area I forms a hydrophobic cluster with nonpolar residues from ηB’-αC loop (Leu369, Trp371) and αC helix (Pro386, Phe387, Phe390) from both protomers. The segment (Asn372-Thr383) between ηB’-αC loop (Leu369, Trp371) and αC helix are disordered in our crystal (dashed lines). (**d**) Contact area II contains salt bridges (Arg365–Glu329) buried inside the interface formed by nonpolar residues of ηA helix (Phe332, Phe333) and β8-α4 loop (His448, Leu451) from both protomers. Hydrogen bonds are indicated by broken lines.

The asymmetric unit contains two PhaC_*Cs*_-CAT molecules (mol A and mol B), which form a face-to-face dimer with a pseudo dyad axis (Fig. 5a). It is likely that the high protein concentrations (~0.3 mM) in our crystallization reproduce the catalytically relevant form in the crystal. It is noteworthy that in the current dimer, the N-terminal ends of both protomers are exposed on the same side of the dimer, implying that the N-terminal subdomain, which is absent in the current PhaC_*Cs*_-CAT, is important for dimer stabilization and that dimerization could be mediated by direct contacts of N-terminal domain from both protomers (Fig. 5b).

In the PhaC_*Cs*_-CAT dimer, the CAP subdomains mediate the intermolecular contacts at the dimer interface, although part of the core domains also participate in formation of the interface. The intimate contact areas comprise ηA (Glu329, Phe332, Phe333) and ηB’(Arg365) helices from each CAP subdomain and the β8-α4 loop (His448, Leu451) from each core subdomain. One of the contact areas (Contact area I) forms a hydrophobic cluster with nonpolar residues from ηB’-αC loop (Leu369, Trp371) and αC helix (Pro386, Phe387, Leu390) from both protomers. The segment (Tyr373-Thr383) between ηB’-αC loop (Leu369, Trp371) and αC helix are disordered in our crystal (dashed lines) (Fig. 5c). Contact area II contains salt bridges (between Arg365 and Glu329) buried inside the interface formed by nonpolar residues of ηA helix (Phe332, Phe333) and β8-α4 loop (His448, Leu451) from both protomers (Fig. 5d). Since β8-α4 loop contains one of the catalytically active residues, Asp447, dimer formation could have some effect on enzymatic activity. The catalytic cysteine residues in the PhaC_*Cs*_-CAT dimer are distant (28.1 Å for the distance between the S_γ_ atoms of Cys residues), and no clear path between them was found in the current structure.

#### Conformational changes in the CAP subdomain induce rearrangement of the dimer interface

**Figure 6:**
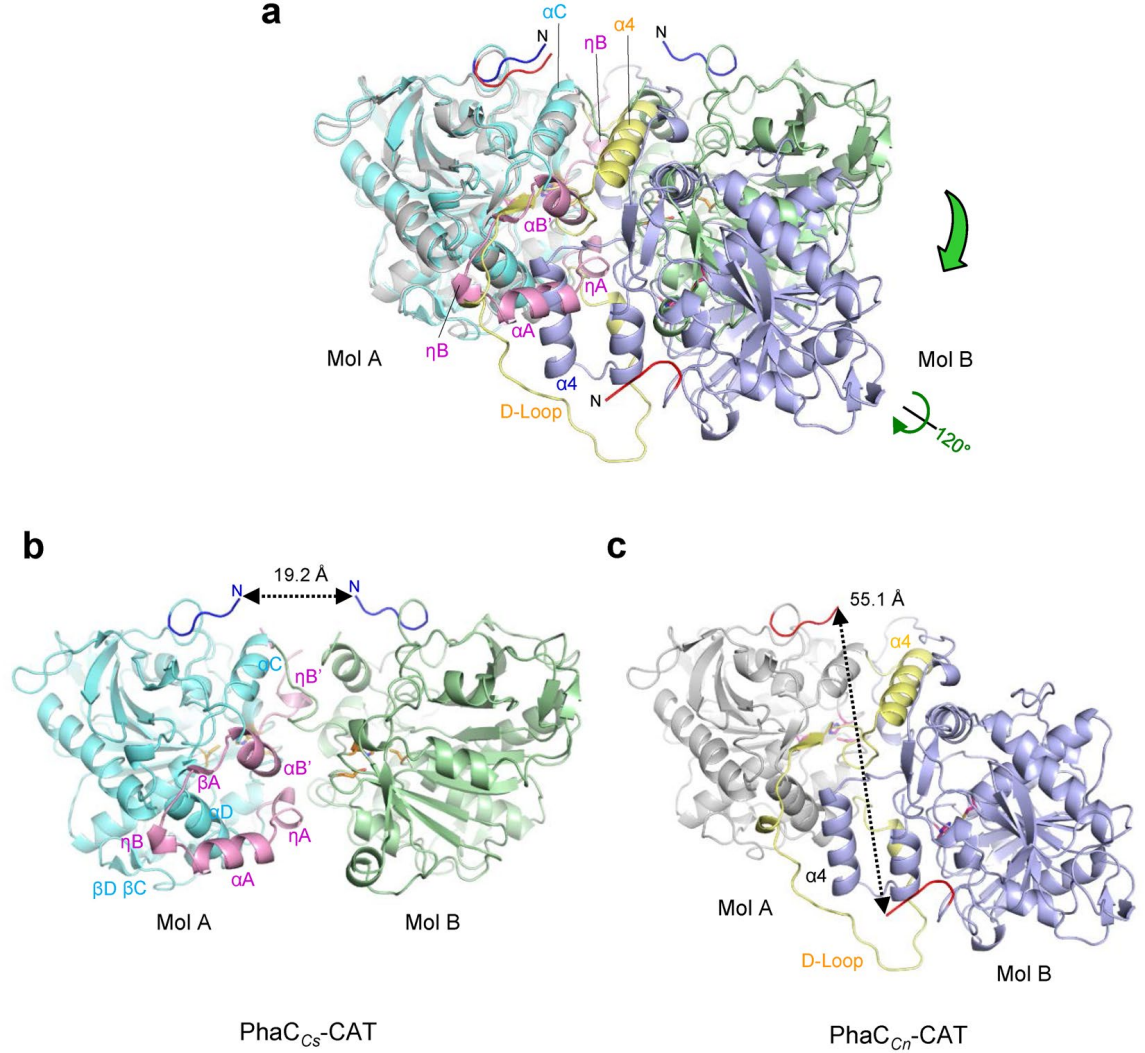
Conformational changes in the CAP subdomain induce dimer organization as revealed by comparison of PhaC_*Cs*_-CAT and PhaC_*Cn*_-CAT dimers. (**a**) Overlay of the PhaC_*Cs*_-CAT dimer (cyan and green) on the PhaC_*Cn*_-CAT dimer (gray and purple). One protomer (cyan) of the PhaC_*Cs*_-CAT dimer is superimposed on one protomer (gray) of the PhaC_*Cn*_-CAT dimer. The distance between the catalytic cysteine residues in the PhaC_*Cs*_-CAT dimer is 28.1 Å, while that in the PhaC_*Cn*_-CAT dimer is 33.3 Å. (**b**) The PhaC_*Cs*_-CAT dimer. The distance between the N-termini is 19.2 Å. (**c**) The PhaC_*Cn*_-CAT dimer with mol A in the same orientation as mol A in the PhaC_*Cs*_-CAT dimer as in (**b**). The dimer interface is reorganized by refolding/unfolding of the LID region with accompanying rotation of one protomer. The distance between the N-termini is 55.1 Å.

Since the CAP subdomain is a major part of the dimer interface, rearrangement of dimer association by conformational changes in the CAP subdomain is evident when comparing the PhaC_*Cs*_-CAT and PhaC_*Cn*_-CAT dimeric structures (Fig. 6). If one protomer of each dimer is superimposed, the other protomer is swung by ~40 Å with a rotation of ~120°. The distance between the catalytic Cys residues in the PhaC_*Cn*_-CAT dimer is 33.3 Å, which is longer than the distance (28.1 Å) in the PhaC_*Cs*_-CAT dimer. The distance between the N-termini of the protomers in the PhaC_*Cn*_-CAT dimer is longer (55.1 Å) than that (19.2 Å) of the PhaC_*Cs*_-CAT dimer, although the N-termini are still located on the same side of the PhaC_*Cn*_-CAT dimer surface (Fig. 6c). This implies that reorganization of the dimer interface induced by refolding/unfolding of the LID region of the CAP subdomain may be facilitated without dissociation of the protomers.

## Discussion

**Figure 7:**
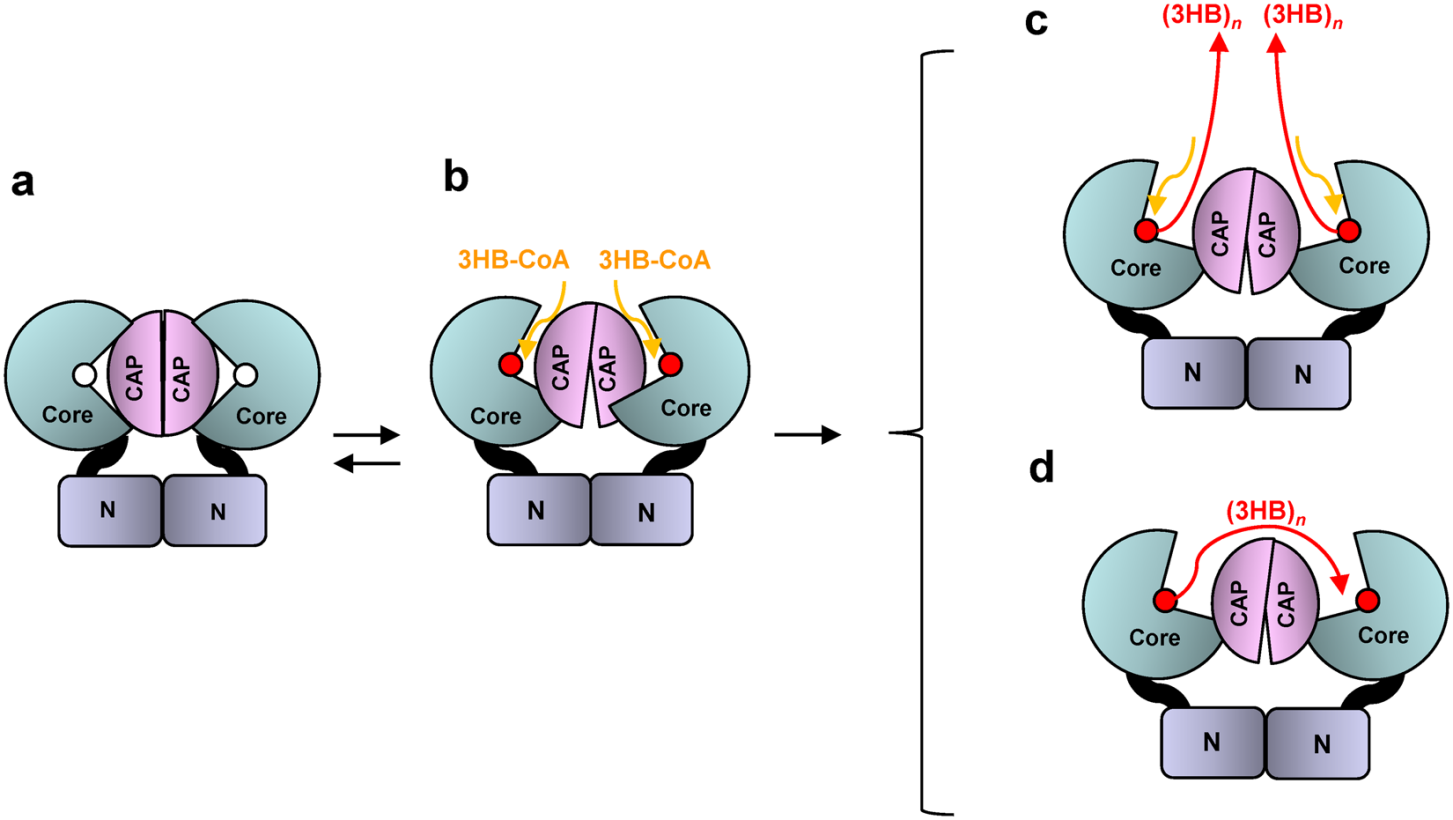
A model of PhaC activation in dimeric form. (**a**) The closed form as observed in our PhaC_*Cs*_-CAT structure. The N domain is proposed to contribute to stabilization of the dimer. (**b**) The partially open form as observed in the PhaC_*Cn*_-CAT structure. The structure provides a possible path to the active site, but no apparent path for product. (**c**) The single active site provides a full active site architecture for initiation of acylation and chain elongation. (**d**) The Cys-bound product in one protomer attacks the 3HB-Cys thioester of the other protomer in the dimer for chain elongation.

We have presented a high resolution structure of PhaC_*Cs*_-CAT. To our surprise the structure of our CAP subdomain was found to differ from that of the recently reported structures of PhaC_*Cn*_-CAT^22, 23^. Inspection of the PhaC_*Cs*_-CAT and PhaC_*Cn*_-CAT structures suggests that the catalytic domain of PHA synthases exists in an open-closed equilibrium in solution by means of conformational changes in the CAP subdomain (Fig. 7a,b). The two reported structures of PhaC_*Cn*_-CAT were obtained from essentially the same crystals produced under similar crystallization conditions using ammonium sulfate at pH 7. These crystals were obtained from crystallization of the full-length protein and found to have a C-terminal catalytic domain by partial degradation during crystallization. Although one of the reported structures is a mutant PhaC_*Cn*_-CAT protein comprising a C319A mutation on the catalytic Cys residue of PhaC_*Cn*_^22^ and displayed a disordered D-loop, the two reported structures display similar partially open form structures, which may be stabilized by an artificial disulfide bond formed between Cys328 and Cys438 of PhaC_*Cn*_-CAT.

The active site architecture of the conserved catalytic triad (Cys-His-Asp) of our structure suggests that His477 is located at a position where the side chain imidazole ring could accelerate deprotonation of the side-chain thiol group of active center Cys291, and nucleophilic attack of the thiol group to the thioester of acyl-CoA would yield the acyl-Cys291 intermediate. In our structure, negatively charged Asp447 assists to enhance the basicity of His477 by formation of a direct hydrogen bond (Fig. 2a). Previous reports suggest that the Asp residue of the triad acts as a general base catalyst to accelerate deprotonation of the 3-hydroxyl group of HB in the step involving elongation of the PHA product^24, 32^. Unfortunately, the precise geometry of this catalytic action of the Asp residue could not be determined from the present structure of the free form.

Two catalytic mechanisms for PHA synthesis have been proposed in the context of dimerization of the synthases^4^. One mechanism is referred to as the non-processive ping-pong model that requires two sets of active sites for PHA chain elongation with chain transfer between the two active center Cys residues across the dimer interface^33, 37^. The second mechanism involves a processive model that requires a single active site for PHA chain elongation and a non-covalent intermediate, in addition to a covalent intermediate bound to the Cys residue at the active center during the catalytic cycle^24, 37–40^. Our dimeric structure shows that the two active sites are too distant (28.1 Å) for successive chemical reactions and seems to favor the mechanism comprising the use of a single active site. A similar conclusion was also discussed previously in relation to a dimeric PhaC_*Cn*_-CAT structure, which shows a long distance (~33 Å) between the active Cys residues^22^ (Supplementary Fig. 5a). In this model, the substrate enters the same substrate-binding tunnel, although chain product is elongated through another path to the protein surface. The first 3HB-CoA forms a 3HB-Cys covalent bond, and the resultant free CoA is released from the tunnel (Supplementary Fig. 5b). The second 3HB-CoA enters the tunnel and attacks the 3HB-Cys thioester bond with the hydroxyl group of the 3HB unit to produce a (3HB)_*2*_-CoA intermediate and free the Cys residue of the active center. This Cys residue is available once again to attack the thioester bond of the (3HB)_*2*_-CoA intermediate to produce (3HB)_*2*_ covalently bound to the Cys residue and release of free CoA from the active site. This cycle is repeated with newly entered 3HB-CoA to produce (3HB)_*n*+*1*_ covalently bound to the Cys residue (Fig. 7c). Thus, the growing 3HB polymer is bound to the enzyme at the end of each cycle, which is consistent with the fact that covalent catalysis occurs using the saturated trimer CoA (sT-CoA), an analogue of (3HB)_*3*_-CoA in which the terminal hydroxyl group is substituted with a hydrogen, which blocks attack by the terminal hydroxyl group^24, 34, 38, 39^.

A slightly different model has also been proposed with essentially the same PhaC_*Cn*_-CAT dimeric structure^23^ (Supplementary Fig. 5c). In this alternative model, two substrates share the same substrate-binding tunnel and the first 3HB-CoA produces 3HB-Cys as in the aforementioned model. The second 3HB-CoA attacks 3HB-Cys to produce (3HB)_*2*_-CoA, which is released from the active site. This cycle is repeated with newly entered 3HB-CoA to produce 3HB-Cys, and then the following (3HB)_*2*_-CoA enters the active site to produce (3HB)_*3*_-CoA, which is again released from the active site. This model is inconsistent with the presence of a stable product bound to the active Cys residue^34^. If the (3HB)_*3*_-CoA that is produced is held in the active site and attacked by the active Cys residue again to produce (3HB)_*3*_-Cys, chain elongation would then require an inter-subunit reaction (Fig. 7d).

The proposed ping-pong mechanism requires two thiol groups located at a distance short enough to shuttle back and forth the growing (3HB)_*n*_ chain between the two thiols. This may be possible if there is another Cys residue near active site Cys291. We found a Cys residue (Cys218) that was located in β4-η1 loop near Site A at the active site (Supplementary Fig. 6a,b). Although Cys218 is conserved in PhaC_*Cn*_ but is not a universally conserved residue, Cys218 may assist in the catalytic action of Cys291 provided that some conformational changes are induced around the active site. However, our mutations involving replacement of Cys218 with Ala or Ser residues resulted in no significant effect on the activity (Supplementary Fig. 6c). Another interesting residue is Thr319 of β7-ηA loop that forms a hydrogen bond (3.5 Å) to Cys291 and is conserved in Class I and IV synthases. Replacement of Thr319 with Ala or Ser, however, had no significant effect on the activity.

**Figure 8:**
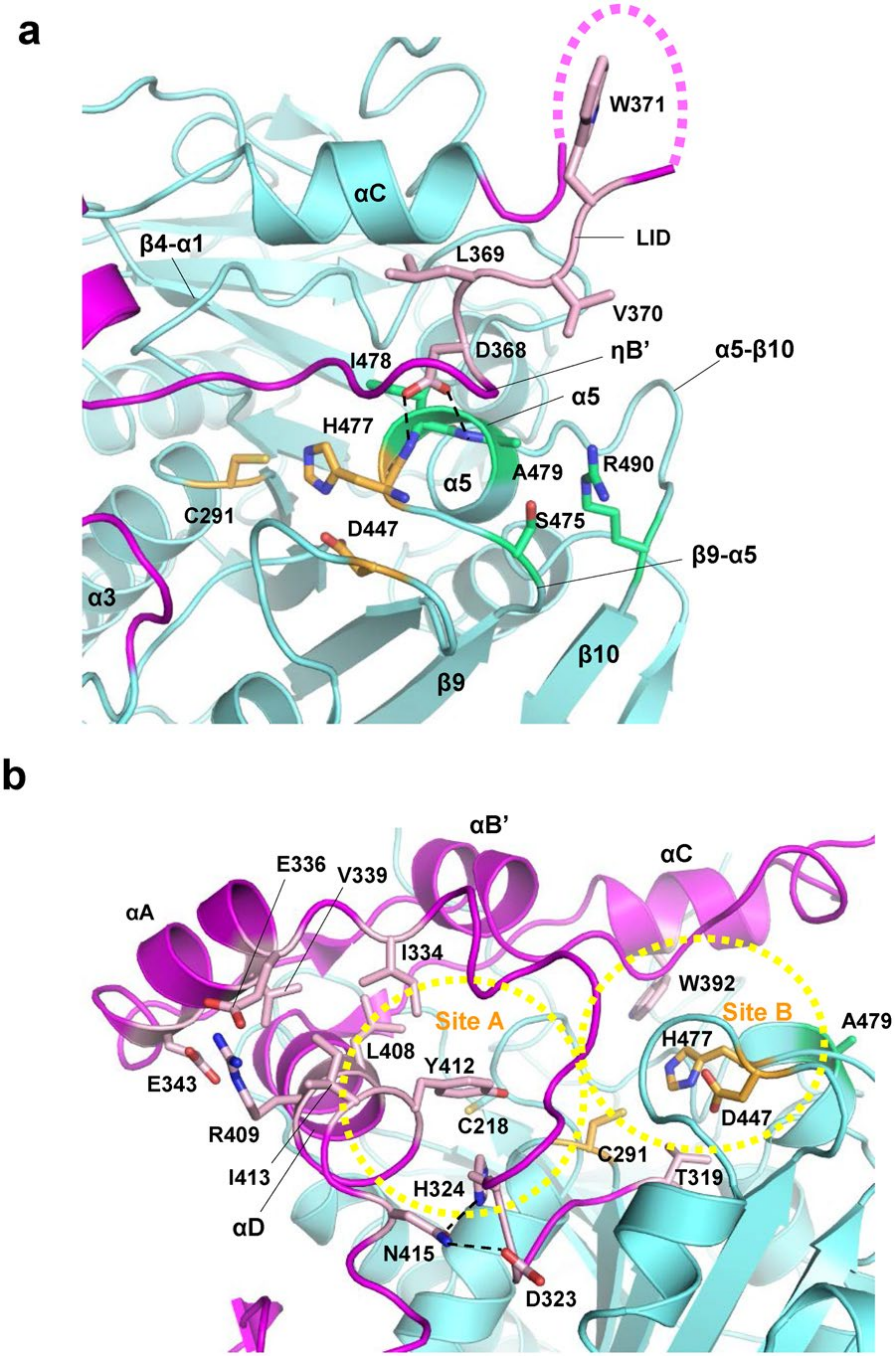
The channel of the active site of PhaC_*Cs*_. (**a**) A close-up view of the β9-α5-β10 segment forming part of the active site of PhaC_*Cs*_-CAT. Ala479 of PhaC_*Cs*_ is located at α5 helix, where His477 of the catalytic triad is also located. Asp368 of the LID region stabilizes α5 helix by forming hydrogen bonds to the main chain amide groups of Ile478 and Ala479 residues which form the helix. Ala479 corresponds to Ala510 of PhaC_*Cn*_. (**b**) A view of the channel at the active site of PhaC_*Cs*_-CAT. Trp392 of PhaC_*Cs*_ is located in the αC helix of the CAP subdomain and faces Site B of the channel where a water cluster is present. Tyr412 and Ile413 are located in the αD helix of the CAP subdomain. Tyr412 projects into Site A of the channel, while Ile413 forms a hydrophobic core with other aliphatic residues.

A previous mutational study of PhaC_*Cs*_ reported that the Class I-conserved residue Ala479 represents at least one of the critical residue required for substrate specificity, as determined by various site-specific mutational assays both *in vivo* and *in vitro*, and production tests of copolymers such as P(3HB-*co*-3HHx)^40^. The greatest enhancement in PHA biosynthesis was observed with the A479G mutation which resulted in a 1.6-fold increase, and mutations of large nonpolar residues (Met, Trp and Val) also enhanced the activity. In our structure, Ala479 is located within α5 helix and the side chain protrudes into a depression of the molecular surface formed by loops (β4-α1, β9-α5 and α5-β10 loops) from the core subdomain, and is partially covered by the 3_10_-helix ηB’ and the following loop of the LID region from the CAP subdomain (Fig. 8a). The side chain of Asp368 of ηB’-helix forms two hydrogen bonds to the main chains of α5 helix. These interactions are typical of N-terminal caps stabilizing the helix, although in this case crowding is induced by the presence of ηB’-helix and the following Val370 residue. Therefore, it was expected that replacement of Ala479 with residues having large side chains would result in the loss of these direct interactions between α5 helix and ηB’-helix. We speculate that this loss results in weakening of the interactions between the LID region and the core subdomain, and stabilizes the active form of this enzyme by releasing the LID region from the active site. Interestingly, a maximum 4-fold increase in 3HHx incorporation was observed for the A479S mutation, while the A479T mutation showed ~2.8-fold enhancement^40^. Since Ala479 is surrounded by polar residues (Ser475 and Arg490), it was reasonable to postulate that replacement of Ala479 with Ser or Thr would facilitate hydrogen-bonding interactions with the polar residues and stabilization of α5 helix harboring the active residue His477, which may be important for enzyme activity.

The CAP subdomain provides αC and αD helices as building blocks of the active site cavity filled with a cluster of water molecules. In our structure, the C-terminal portion of the LID region of the CAP subdomain is disordered and is followed by αC helix docked to the core subdomain. Two highly conserved residues, Trp392 and Asp395, are present in αC helix (Supplementary Fig. 1). Asp395 stabilizes αC helix docking by forming a salt bridge with highly conserved Lys221 (β4- η1 loop) of the core subdomain. Trp392 is part of the group of residues forming the active site and stabilizes the αC helix by anchoring the nonpolar side chain to the nonpolar site (Fig. 8b). Since αC helix is important for dimerization (Fig. 5), mutation of Trp392 resulted in disruption of dimerization of PhaC_*Cn*_ (Trp425 in PhaC_*Cn*_)^18^ and abolished the activity of Class II PhaC1 from *Pseudomonas aeruginosa* (Trp398 in PhaC1_*Pa*_)^26^. At the channel of the active site, αD helix is the building block of Site A (Fig. 8b). Tyr412 and Ile413 of αD helix form a hydrophobic core together with other nonpolar residues from αD helix (Met407 and Leu408) and αA helix (Ile334 and Val339). Mutations of residues corresponding to Tyr412 and Ile413 in PhaC_*Cn*_ resulted in a reduction in enzyme activity^41^. The C-terminal end of this helix possesses highly conserved Asn415, which forms hydrogen bonds with β7-ηA loop (Asp323 and His324), and contributes to formation of the Site A channel. As described above, Tyr412 and His324 are polar residues facing the channel and may participate in substrate binding. It is noteworthy that Tyr412 is conserved in Class I, III and IV PHA synthases, which utilize SCL substrates. In contrast, Class II synthases, which utilize MCL substrates, have a preference for Phe at this position. These differences require further experimental investigation.

A study employing random mutagenesis revealed a beneficial mutation (F518I) of PHA synthase from *Aeromonas punctata* (PhaC_*Ap*_)^42^. Compared with wild-type PhaC_*Ap*_, the F518I mutation in PhaC_*Ap*_ (equivalent to Tyr492 in PhaC_*Cs*_) increased the relative activity to 480% in an *in vitro* synthase activity assay and 120% in terms of *in vivo* PHA accumulation ability. In our structure, Tyr492 is located within β10 strand and stabilizes β-sheet formation between β10 and β11 strands by engaging in an aromatic stacking interaction with adjacent His512 from β11 strand (Supplementary Fig. 7). Formation of a hydrogen bond between Thr494 and His512 also contributes to stabilization of the β-sheet. Multiple sequence alignments revealed a similarity in aromatic amino acids at the position corresponding to Tyr492 of PhaC_*Cs*_ in Class I and II synthases. However, the residues in these positions differ between PhaC_*Ap*_ and PhaC_*Cs*_, where residues Tyr492, Thr494 and His512 (PhaC_*Cs*_) are replaced with residues Phe518, His520 and Gln538 (PhaC_*Ap*_), respectively. In PhaC_*Ap*_, Phe518 and His520 are stacked, and Gln538 and His520 may form a hydrogen bond which stabilizes the β-sheet. The F518I mutation may improve hydrocarbon chain interactions between Gln538 and Ile518. Since catalytic residue His477 is located at the N-end of α5 helix, which is connected to strand β10, fine-tuning of this catalytic residue by the F518I mutation in PhaC_*Ap*_ may have resulted in the enhanced activity. Further analysis should be employed to clarify the details of the mechanism of the enhanced activity associated with the F518I mutation. Comparison of the primary sequences of Class I PhaC_*Cs*_ and Class II PhaC1 revealed the presence of conserved residues comprising tyrosine, threonine and histidine, suggesting that these PHA synthases may share the same architecture in maintaining the β strands observed in the current structure.

A previous study showed that the F420S mutation in PhaC_*Cn*_ increased the specific activity with a significantly reduced lag phase^43^. This residue corresponds to Phe387 of PhaC_*Cs*_, which is conserved among Class I and II PHA synthases, and is located in αC helix of the CAP domain (Fig. 5c). As previously discussed, Phe387 is involved in dimerization by participating in an intermolecular nonpolar interaction directly connected to the LID region, suggesting that the mutation may contribute to a reduction in the lag phase by affecting the conformational stability and/or conformational transition of the LID region. The conformational characteristics of the LID region should be important for the enzymatic activity. A previous study utilizing random mutagenesis found that A391T and T393A mutations in PhaC_*Cn*_ (corresponding to G358 and T360 located in αB’ helix of the LID region in PhaC_*Cs*_) resulted in synthase activity at lower efficiency^44^. In the recently reported structures of PhaC_*Cn*_-CAT^22, 23^, Ala391 and Thr393 are located at the interface between the LID region and the core subdomain, and are engaged in nonpolar and polar interactions, respectively. In our PhaC_*Cs*_-CAT structure, G358 and T360 interact with another part of the LID region rather than the core subdomain. These residues are not necessarily conserved in other synthase classes (Supplementary Fig. 1) and may reflect the characteristic properties of each enzyme. However, the conformational properties of the LID region are affected by the component residues interacting with other parts of the enzyme and have some effect on the catalytic activity.

In conclusion, we have defined a closed form of the catalytic domain of PhaC_*Cs*_, a Class I PhaC. The catalytic site is covered by the CAP subdomain and the catalytic residues are facing a water-filled large channel inside the protein. This form displays a sharp contrast to the recently reported structure of the partially open form of the catalytic domain of PhaC_*Cn*_. The major difference between the closed and partially open forms is found in the conformation of the CAP subdomain. Both catalytic domains of PhaC_*Cs*_ and PhaC_*Cn*_ form a dimer mediated by the CAP subdomain. Therefore, the arrangement of the protomers to form a dimer also differs in these two forms. These facts suggest that the CAP subdomain should undergo a conformational change during catalytic activity with rearrangement of the dimeric form. Detailed information concerning the three-dimensional structure of the catalytic domain provides valuable clues to delineate the obscure catalytic action involved, and should assist in efforts to improve the functionality and activity of this industrially important enzyme.

## Materials and Methods

### Cloning, expression and purification

The gene of full-length PHA synthase *phaC*_*Cs*_ (1704 nucleotides) and the catalytic domain *phaC*_*Cs*_ *-CAT* (1179 nucleotides) from *Chromobacterium* sp. USM2 (JCM15051, RIKEN BRC) were cloned from the bacterial genome and inserted into His-fusion vector pET47b [+] (Novagen). The integrity of the coding region was verified by DNA sequencing. The plasmid was transformed into *Escherichia coli* Rosetta 2 (DE3) (Novagen) and cells were grown in LB medium supplemented with 50 µg/ml Kanamycin and 35 µg/ml Chloramphenicol at 37 °C until the OD_600nm_ reached a value of 0.6. Protein expression was induced by the addition of isopropyl-β-D-thiogalactoside (IPTG) to a final concentration of 100 µM. The culture was induced for another 20 hours at 20 °C and cells were harvested by centrifugation. The cells were then suspended in 2X PBS (phosphate-buffered saline) with 3 mM β-mercaptoethanol (β-ME) and disrupted by sonication in an ice bath. The soluble fraction was separated by ultracentrifugation and then loaded onto a Ni-NTA agarose column (QIAGEN). The column was washed with buffer containing 20 mM Tris-HCl (pH 8.0), 100 mM NaCl, 3 mM β-ME and 10 mM Imidazole, and target protein was eluted using the same buffer containing 250 mM Imidazole *in lieu* of 10 mM Imidazole. The column effluent was loaded onto a HiTrap Q anion-exchange column (GE Healthcare) and eluted using a gradient of 0-500 mM NaCl in buffer containing 20 mM Tris-HCl (pH 8.5) and 3 mM β-ME. The eluted products were pooled and digested overnight at 4 °C using PreScission protease (GE Healthcare). The digested product was loaded onto a Ni-NTA agarose column to remove the His-tag and co-purified products. Flow-through from the column was collected and concentrated by centrifugation using an Amicon Ultra 30,000 molecular weight cut-off filter (Merck Millipore). The concentrated product was finally passed through a Superdex 200 gel filtration column (GE Healthcare) using buffer comprising 10 mM Tris-HCl (pH 8.0), 100 mM NaCl and 3 mM β-ME. Peak fractions were collected and concentrated to about 50 mg/ml. SDS-PAGE of the protein samples gave one major band corresponding to ~63 kDa (full-length PhaC_*Cs*_) and ~43 kDa (PhaC_*Cs*_-CAT). Analysis of the sample using matrix-assisted laser desorption/ionization time-of-flight mass spectrometry (MALDI-TOF MS; Bruker Daltonics) confirmed that the target proteins were successfully purified without degradation. Two additional vector-derived residues (Gly-Pro) were identified at the N-terminus. Samples were frozen in liquid nitrogen and stored at −80 °C until use. Selenomethionine (SeMet)-labeled full-length PhaC_*Cs*_ was expressed in M9 medium containing SeMet under conditions that inhibited the methionine biosynthetic pathway. The procedures employed for protein expression and purification were the same as described above, except that dithiothreitol (5 mM) was used *in lieu* of β-ME. Analysis using MALDI-TOF MS confirmed that all twenty-one methionine residues in PhaC_*Cs*_ had been successfully replaced with SeMet.

### Crystallization

Preliminary crystallization screening was performed using the vapor-diffusion method at both 4 °C and 20 °C using commercially available screening kits (Hampton Research and QIAGEN). Protein (0.3 mM) was mixed at 1:1 ratio with the reservoir solution (Supplementary Fig. 8). Crystals of PhaC_*Cs*_ were observed after one year in equilibrium against mother liquor containing 0.2 M lithium sulfate, 0.1 M Bis-Tris (pH 6.5) and 25% PEG3350 (Hampton Research) at 20 °C. Crystals were cryoprotected by the addition of 20% glycerol and flash-cooled using liquid nitrogen. However, the production of these tiny hexagonal crystals (<100 μm) could not be repeated using the original kit reagent or self-made mother liquor. In efforts to reproduce the crystals, full-length PhaC_*Cs*_ was successfully crystallized in the presence of α-chymotrypsin protease at a ratio of 1 µg protease to 600 µg PhaC_*Cs*_^45^. Crystals of α-chymotrypsin-digested PhaC_*Cs*_ were grown after four days *in lieu* of one year under the same conditions. The crystals were cryoprotected in 0.1 M Bis-Tris (pH 6.5), 0.2 M lithium sulfate, 15% PEG3350 and 33% ethylene glycol and flash-cooled using liquid nitrogen. The crystals of α-chymotrypsin-treated SeMet-labeled PhaC_Cs_ were optimized by homo- and hetero-seeding under the same conditions as described above. Crystals of PhaC_*Cs*_-CAT were grown in 0.2 M ammonium sulfate and 15% PEG4000 at 4 °C after 20 days. The crystal was optimized by streak-seeding in 50 mM Bis-Tris (pH 5.5), 60 mM ammonium sulfate and 5% PEG4000. The crystal was cryoprotected in 33% ethylene glycol.

### X-ray data collection, phasing and refinement

All X-ray data were collected at SPring-8, Harima, Japan. During X-ray beam exposure, crystals were flash-cooled and maintained at 100 K using a nitrogen stream. Detailed statistics of the structure determination are shown in Supplementary Table 1. All diffraction data were indexed and merged using the DENZO and SCALEPACK programs included in HKL2000^46^. Molecular replacement using *in silico*-predicted structures of PhaC_*Cs*_ (N.F. Jamil, unpublished), PhaC_*Cn*_ (S.X. Choong, unpublished) and the crystal structure of dog gastric lipase (PDB code 1K8Q) as reference models was failed.

The phase problem of PhaC_*Cs*_ was solved by single-wavelength anomalous dispersion (SAD) using an α-chymotrypsin-digested SeMet-labeled PhaC_*Cs*_ crystal. Determination of the selenium positions, phasing, density modification, and initial modeling of the SeMet-labeled PhaC_*Cs*_ structure were performed using the autoSHARP program suite^47^ comprising the heavy atom search program ShelxC/D^48^, the phasing program SHARP^47^, and density modification program SOLOMON^49^. Buccaneer^50^ and ARP/wARP^51^ were then employed to build the model automatically. Subsequent manual model building and refinement were performed using refmac5^52^, phenix.refine^53^ and Coot^54^. Other structures were determined by molecular replacement using the Phaser program^55^. Finally, model structures were further refined using refmac5, phenix.refine and Coot. Superposition of the PhaCs and lipases was performed using the program LSQKAB^56^. Illustrations were prepared using the program PyMOL (DeLano Scientific).

### Analytical centrifugation (AUC)

Sedimentation velocity ultracentrifugation experiments were performed using a Beckman Coulter Optima XLA analytical ultracentrifuge at 20 °C. Full-length, PhaC_*Cs*_ (calc. 63.4 kDa) samples were dissolved in 10 mM Tris-HCl (pH 8.0), 100 mM NaCl and 3 mM β-ME at a concentration of 5 µM in the absence or presence of 50 µM DL-3HB-CoA and centrifuged at 20,000 rpm. Similary, PhaC_*Cs*_-CAT (calc. 43.8 kDa) samples were prepared at a concentration of 5 or 20 µM in the absence of DL-3HB-CoA. The raw results were analyzed using the program SEDFIT.

### Size exclusion chromatography (SEC)

Analytical gel filtration was performed with protein samples in the absence or presence of substrate by loading onto a Superdex 200 (10/30) gel filtration column in buffer containing 10 mM Tris-HCl (pH 8.0), 100 mM NaCl and 3 mM β-ME at 4 °C. A final concentration of 30 µM protein in the absence or presence of 0.5 mM DL-3HBCoA was loaded and its elution profile was compared with the standard elution profile. The molecular weights of the eluted peaks were calculated and compared with monomeric standard molecular weights.

### *In vitro* enzymatic assay

PHA synthase activity assays were performed by measuring the CoA released from the substrates during the polymerization process^21^. N-terminal His-tag fused PhaC_*Cs*_-C291A, PhaC_*Cs*_-D447A and PhaC_*Cs*_-H477A were used in the enzyme assay for catalytic triad verification. The enzyme assay was performed at 30 °C in a final volume of 360 µl reaction mixture containing 30 mM potassium phosphate buffer (pH 7.8), 1 mg/ml BSA, 0.6 mM DL-3HB-CoA (Sigma Aldrich), and 1 mM 5,5-dithio-bis-(2-nitrobenzoic acid). Following the addition of enzymes (7.5–30 nM) into the reaction mixture, measurement of CoA release was taken at various time points by measuring the absorbance at 412 nm using a UV spectrophotometer (Shimadzu Spectrophotometer UV-1800)^33^. The activities of individual mutants of PhaC_*Cs*_-C218A, C218S, T310A and T310S were examined under same conditions except the phosphate buffer was replaced with 20 mM Tris-HCl (pH 7.5).

### *In vivo* enzymatic assay

The catalytic triad mutant *phaC*_*Cs*_ was amplified using forward and reverse primers with additional 5′-*Xba* I and 3′-*Eco* RI restriction recognition sites. PCR templates were based on pCold-C291A, pCold-D447A and pCold-H477A. All three amplicons were inserted into pYEB-100 plasmid (B.A. Ghani, unpublished), transformed into *E. coli* S17–1 λpir, and subsequently conjugated with *C. necator* H16 strain PHB^−^4 (PHA-negative mutant of H16).

All *C. necator* H16 mutants PHB^−^ harboring wild-type and catalytic triad mutant plasmids were grown in mineral medium supplemented with 0.5% crude palm kernel oil (CPKO) as the carbon source for 48 hours in a 30 °C incubator with shaking. The four cultures in this study comprised PHB^−^4-PhaC_*Cs*_, PHB^−^4-C291A, PHB^−^4-D447A and PHB^−^4-H477A. Cells were harvested and isolated polymer was subjected to analysis by gas chromatography (GC) as follows. Methanolysis of lyophilized cells in solution containing 85% (*v/v*) methanol and 15% (*v/v*) sulfuric acid was performed at 100 °C for 140 minutes to convert monomeric constituents to their hydroxyacyl methyl esters^57^. Then, 1 ml of distilled water was added to each tube and vortexed vigorously. The tubes were then allowed to stand for 5 minutes to facilitate phase separation. The lower chloroform phase containing the hydroxyacyl methyl ester was transferred into another tube containing anhydrous sodium sulfate anhydrous to remove traces of water. An aliquot of 500 µl of hydroxyacyl methyl ester solution were pipetted into autosampler vials containing 500 µl of caprylic acid methyl ester (CME) as an internal standard. The prepared hydroxyacyl methyl ester samples were analyzed using a GC machine (SHIMADZU Gas Chromatograph GC-2010). Internal standard CME was prepared by the addition of 1.0 ml of caprylate methyl esters to 499.0 ml of chloroform. The carrier gas used was nitrogen with a hydrogen flame serving as the Ionization Detector.

### Data Availability

Atomic coordinates and structure factors for PhaCCs-CAT have been deposited in the Protein Data Bank under ID code 5XAV.

## Electronic supplementary material

Supplementary figures
