# Supplementary material for: Structure of polyhydroxyalkanoate (PHA) synthase PhaC from Chromobacterium sp. USM2, producing biodegradable plastics
Source: Sci Rep. 2017 Jul 13;7:5312. doi: 10.1038/s41598-017-05509-4 (PMC5509742; doi:10.1038/s41598-017-05509-4)
Supplement: Supplementary file 1 — Supplementary figures [file 41598_2017_5509_MOESM1_ESM.pdf]

Structure of polyhydroxyalkanoate (PHA) synthase PhaC from *Chromobacterium* sp. USM2, producing biodegradable plastics

Min Fey Chek[1], Sun-Yong Kim[1], Tomoyuki Mori[1], Hasni Arsad[2], Mohd. Razip Samian[3], Kumar Sudesh[3] and Toshio Hakoshima[1]

[1] Structural Biology Laboratory, Nara Institute of Science and Technology, 8916-5 Takayama, Ikoma, Nara 630-0192, Japan
[2]Advanced Medical and Dental Institute, Universiti Sains Malaysia, Bertam 13200, Kepala Batas, Penang, Malaysia
[3] School of Biological Sciences, Universiti Sains Malaysia, 11800 Penang, Malaysia

* Corresponding author:

Supplementary Table 1. Crystallographic statistics of the catalytic domain of PhaC$_{Cs}$-CAT

Crystallographic Analysis Statistics		
Crystal form	Native	SeMet (SAD)[a]
Space group	$P6_1$	$P6_2$
Unit cell		
a , *b* , *c* (Å)	117.33, 117.33, 105.91	117.15, 117.15, 52.69
α, *β*, *γ* (°)	90, 90, 120	90, 90, 120
Wavelength (Å)	0.90000	0.97906 (Se peak)
Resolution range[b] (Å)	50.00 - 1.48 (1.53 - 1.48)	50.00 - 2.50 (2.54 - 2.50)
Reflections[c]		
Oscillation range (°)	360	360
Measured/Unique	1535,125/137,720	304,374/14,563
Multiplicity	11.2 (11.2)	20.9 (22.5)
Mosaicity (°)	0.17 – 0.34	0.44 - 0.73
I/s(*I*)	58.6 (6.2)	73.5 (15.5)
R_{merge}(%)	7.5 (64.4)	12.2 (76.4)
Completeness (%)	99.8 (100.0)	100.0 (100.0)
Number of heavy atoms[d]	-	5
FOM (acentric/centric)[e]	-	0.365/0.161

Refinement Statistics	
R_{work} / R_{free} (%)[f]	12.11/15.74
Number of atoms	6968
Protein molecules	2 (754 residues)
Water molecules	1050
Average B-factors (Å^2)	
Protein	20.1
Water molecules	33.5
R.m.s.d. from ideal values	
Bonds (Å)/ angles (°)	0.019/1.603
Ramachandran plot (%)	
Favored	97.0
Allowed	2.7
Outliers	0.4

[a] The SeMet derivative was treated with α-chymotrypsin.
[b] Values in parentheses are for the highest-resolution shell.
[c] collected at SPring-8 beamline BL44XU with a MX300HE detector (each 1° oscillation) at 100°K.
[d] Heavy atoms were searched with the program, SHELX C/D.
[e] The figure of merit (FOM) was calculated with the program, SHARP/autoSHARP.
[f] R_{free} was calculated on a random 5 % reflections of the data.

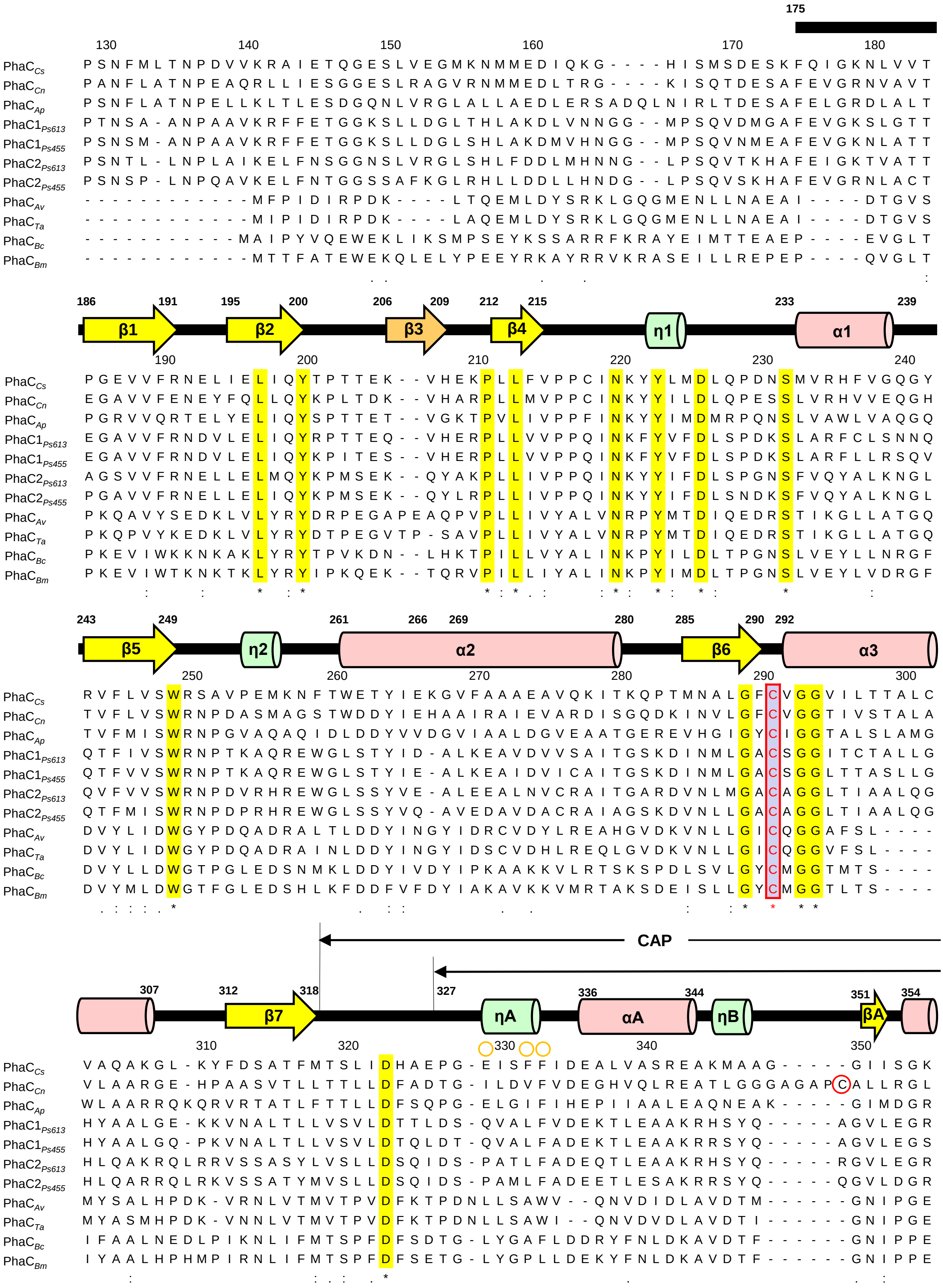

Supplementary Figure 1

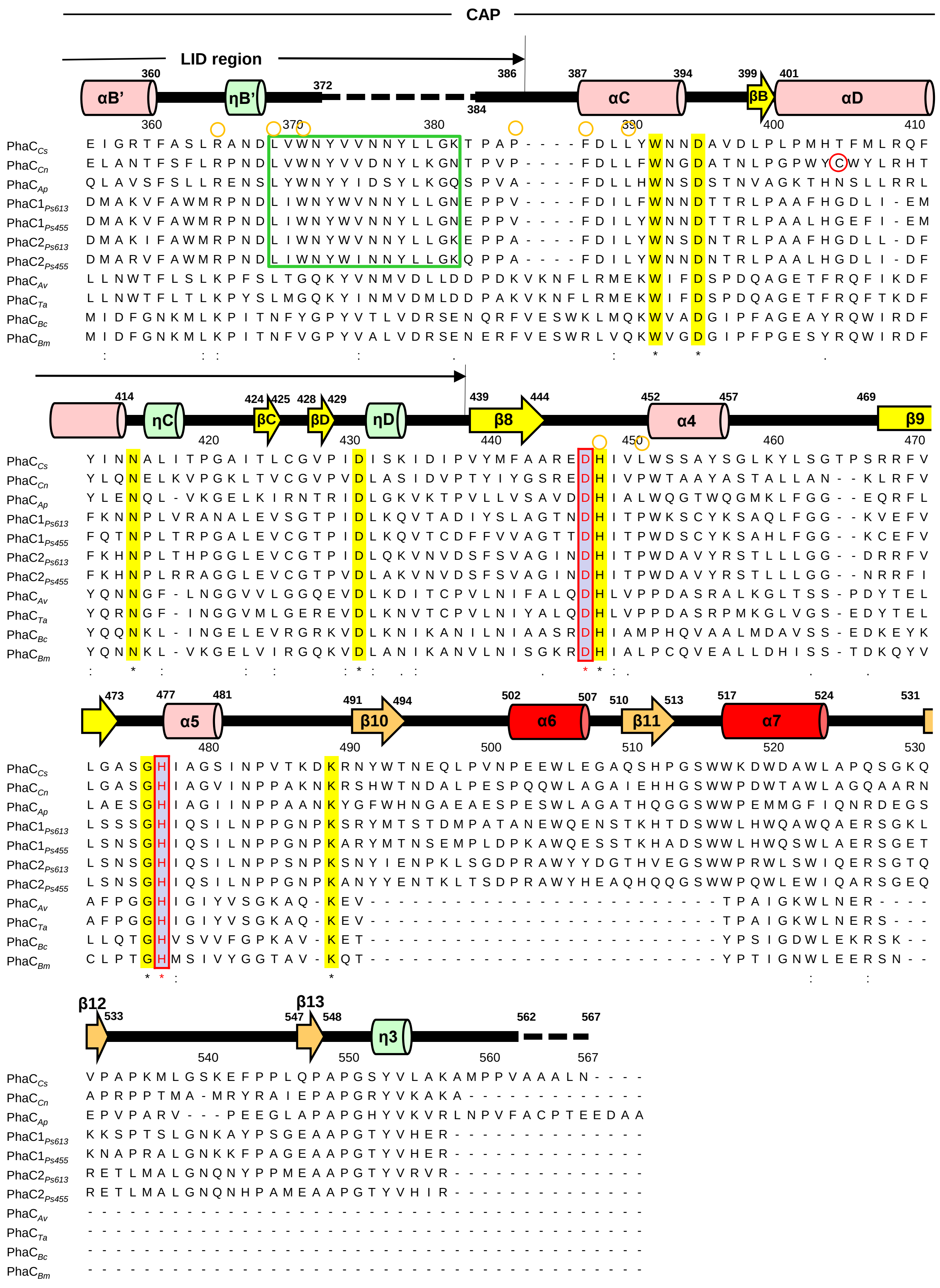

Supplementary Figure 1 (continued)

Supplementary Figure 1
Alignment of amino acid sequences of PhaC catalytic domains.

The secondary structure elements found in the structure of PhaC$_{Cs}$-CAT are shown at the top of the alignment with α-helices (pink cylinders), β-strands (yellow arrows), loops (bold lines) and missing loops (broken lines). Glu329, Phe332, Phe333, Arg365, His448 and Val450 of PhaC$_{Cs}$-CAT are marked with orange circles at the top. Two Cys residues (Cys382 and Cys438 of PhaC$_{Cn}$-CAT) forming a disulfide bond are marked by red circles. Part (Leu402–Asn415 of PhaC$_{Cn}$-CAT) of the LID region forming α4 helix in the structure is conserved in members of Class I and II synthases. These sequences are marked by circles within a green box.

The abbreviations are as follows;

Class I	PhaC$_{Cs}$:	*Chromobacterium* sp. USM2 (ADL70203)
	PhaC$_{Cn:}$	*Cupriavidus necator* (AAW65074)
	PhaC$_{Ap}$:	*Aeromonas punctata* (BAA21815)
Class II	PhaC1$_{Ps61\text{-}3}$:	*Pseudomonas* sp. 61-3 (BAA36200)
	PhaC2$_{Ps61\text{-}3}$:	*Pseudomonas* sp. 61-3 (BAA36202)
	PhaC1$_{PsUSM4\text{-}55}$:	*Pseudomonas* sp. USM4-55 (ABX64434)
	PhaC2$_{PsUSM4\text{-}55}$:	*Pseudomonas* sp. USM4-55 (ABX64435)
Class III	PhaC$_{Av}$:	*Allochromatium vinosum* DSM180 (BAE20055)
	PhaC$_{Tv}$:	*Thiocystis violascens* DSM198 (AFL75311)
Class IV	PhaC$_{Bc}$:	*Bacillus cereus* (BAI68395)
	PhaC$_{Bm}$:	*Bacillus megaterium* (AAD05260)

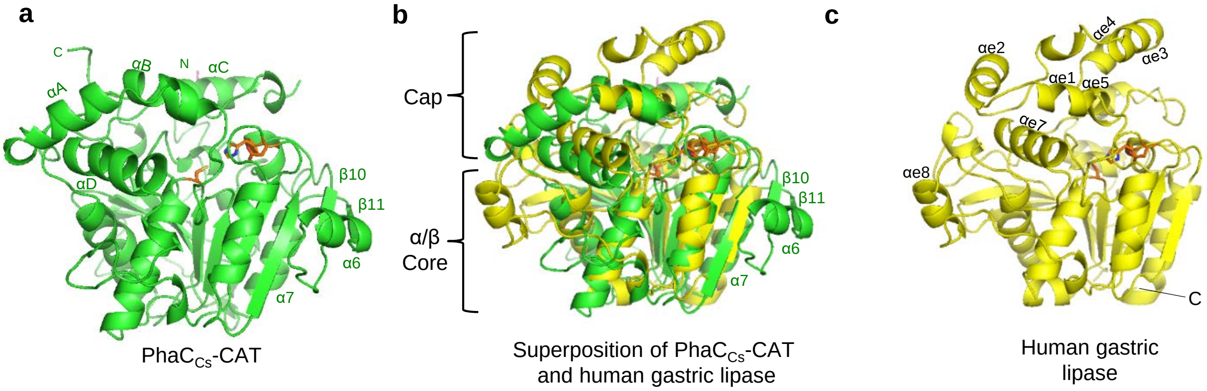

Supplementary Figure 2
Structural comparison of the catalytic domains of PhaC$_{Cs}$ and lipase.

(**a**) A side-view of PhaC$_{Cs}$-CAT determined in the current study.
(**b**) As in **a**, but overlaid on human gastric lipase. The α/β core subdomains are well overlapped with a small r.m.s. deviation (1.02 Å), whereas the CAP subdomains are poorly overlapped. The α/β core subdomain of PhaC$_{Cs}$-CAT contains an additional segment comprising β10-α6-β11-α7.
(**c**) A side-view of human gastric lipase (PDB code 1HLG).

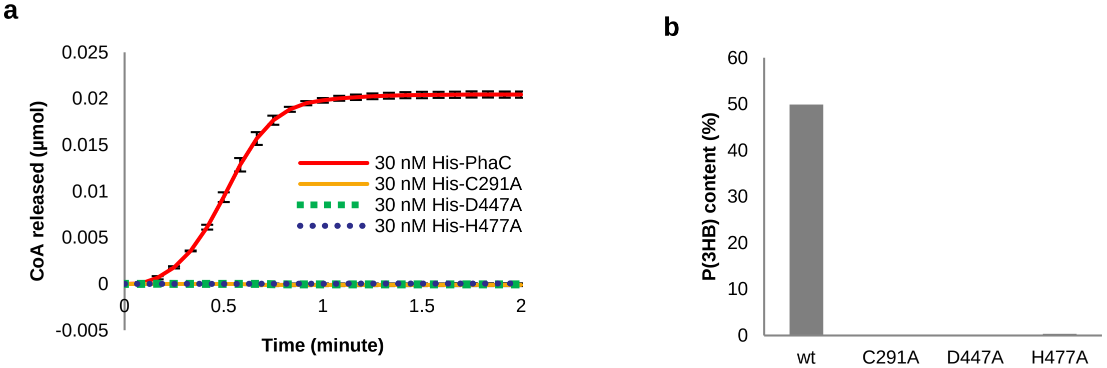

Supplementary Figure 3
Verification of catalytic triad of PhaC$_{Cs}$

(**a**) *In vitro* PHA synthase activity of wild-type and mutant PhaC$_{Cs.}$(full-length). Wild-type PhaC$_{Cs}$ showed higher activity compared with the three catalytic mutants – C291A, D447A and H477A. CoA released from the enzymatic reaction by wild-type N-terminal His-tagged fusion PhaC$_{Cs}$ was detected and reached its maximum consumption of substrate within 1 minute, whereas no significant free CoA was detected from the other three individual catalytic triad mutants.
(**b**) *In vivo* poly(3-hydroxybutyrate) [P(3HB)] production in wild-type and mutant full-length PhaC$_{Cs}$ using 0.5% CPKO as carbon source. Wild-type PhaC$_{Cs}$ (wt) accumulated P(3HB) at 49.9wt% while the accumulation of P(3HB) of mutant PhaC$_{Cs}$ with C291A, D447A, and H477A are 0.1, 0.1, and 0.4wt%, respectively.

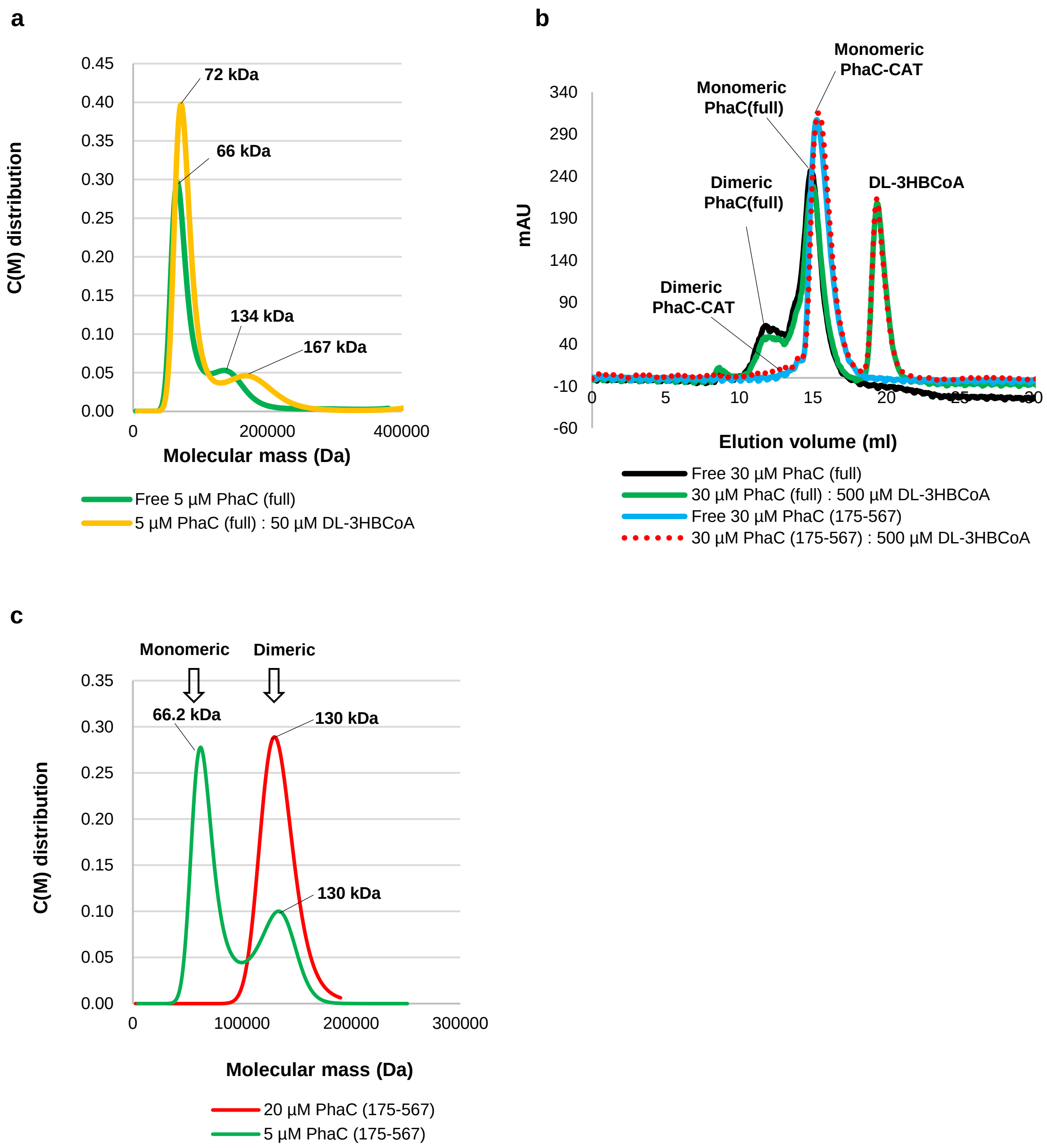

Supplementary Figure 4
Oligomerization equilibrium of PhaC in solution.

(**a**) Analytical ultracentrifugation (AUC) analyses of PhaC$_{Cs}$ (full-length, 63.4 kDa) using a sedimentation velocity method. The equilibria between monomeric and dimeric forms in solution were observed in the absence or presence of substrate (DL-3HB-CoA). The monomeric form is dominant at 5 µM PhaC$_{Cs}$ in 10 mM Tris-HCl (pH 8.0), 100 mM NaCl and 3 mM β-ME at 20˚C, with a small portion being present in the dimeric form. In the presence of substrate, a higher (presumably tetrameric) form appears.
(**b**) Size exclusion chromatography (SEC) of PhaC$_{Cs}$ (full-length) and PhaC$_{Cs}$(175-567), which is PhaC$_{Cs}$-CAT, in the absence or presence of substrate DL-3HB-CoA. The elution profiles of PhaC$_{Cs}$ and PhaC$_{Cs}$-CAT were compared.
PhaC$_{Cs}$ (full-length) exists in monomeric and dimeric (dominant) forms. Addition of DL-3HB-CoA induced only a small change with the appearance of a faint peak, presumably that of the tetrameric form. In contrast to these equilibria, PhaC$_{Cs}$-CAT exists in a monomeric form at a concentration of 30 µM both in the absence or presence of substrate.
(**c**) AUC analyses of PhaC$_{Cs}$-CAT using a sedimentation velocity method. The dimeric form of PhaC$_{Cs}$-CAT was detected at 5 µM (green) and significantly stabilized at a high concentration (20 µM, red).

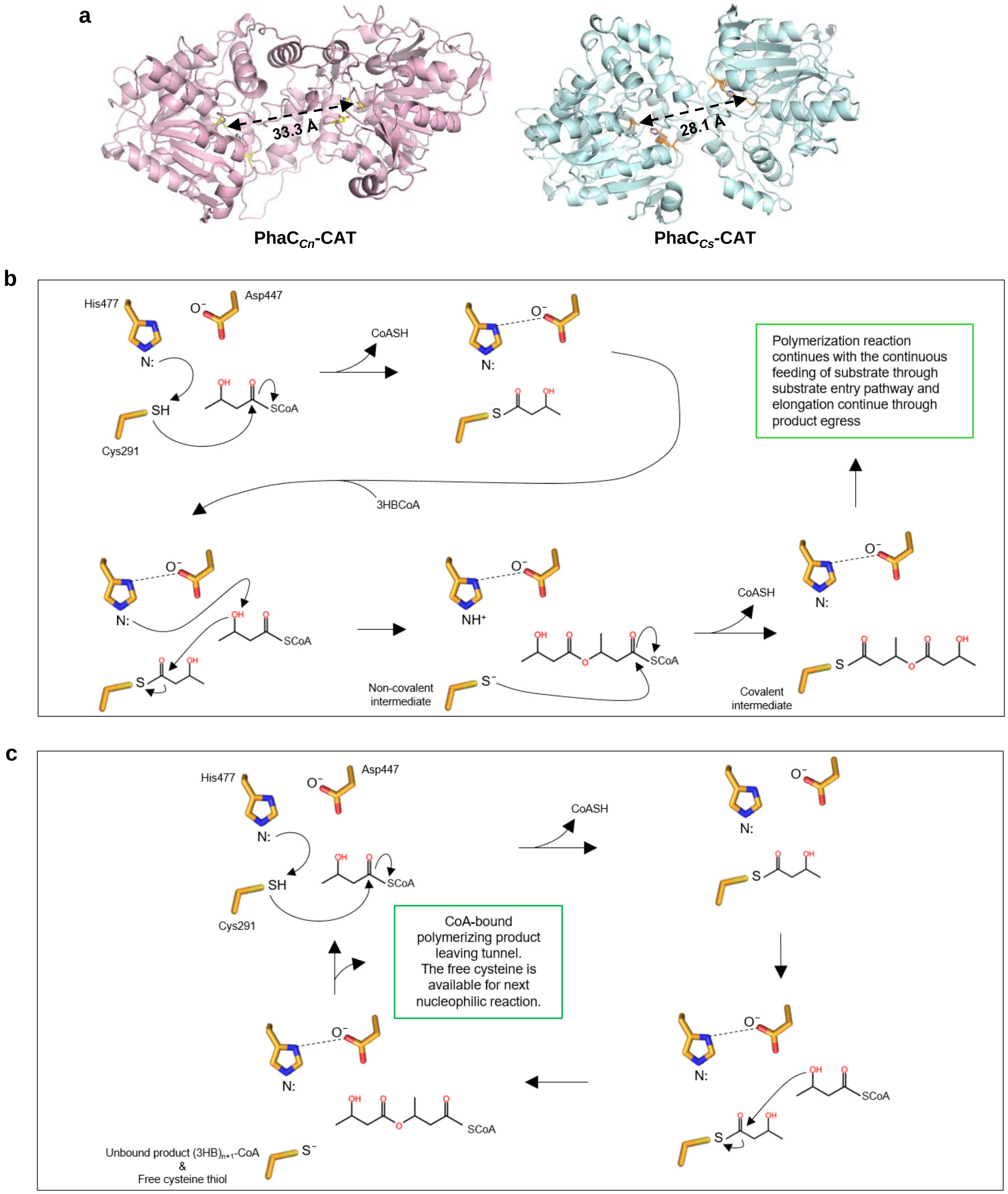

Supplementary Figure 5

Proposed mechanism

(**a**) The distance of the catalytic cysteines from both protomers. The distance (broken lines) between the S_γ from both mol A and mol B is 33.3 Å in dimer PhaC$_{Cn}$-CAT (5HZ2)[23] (*left*) and 28.1Å in dimeric PhaC$_{Cs}$-CAT (*right*).

(**b**) Processive single active site model (In and out tunnels) which is also proposed in reference[22]. This model requires a single active site for PHA chain elongation and a non-covalent intermediate, in addition to a covalent intermediate bound to the Cys residue at the active center during the catalytic cycle.

(**c**) Alternative processive single active site model (Single tunnel) which is proposed in reference[23]. In this model, two substrates share the same substrate-binding tunnel and the first 3HB-CoA produces 3HB-Cys. The second 3HB-CoA attacks 3HB-Cys to produce (3HB)$_2$-CoA, which is released from the active site. The cycle is repeated with newly entered 3HB-CoA to produce 3HB-Cys, and then the following (3HB)$_2$-CoA enters the active site to produce (3HB)$_3$-CoA, which is again released from the active site.

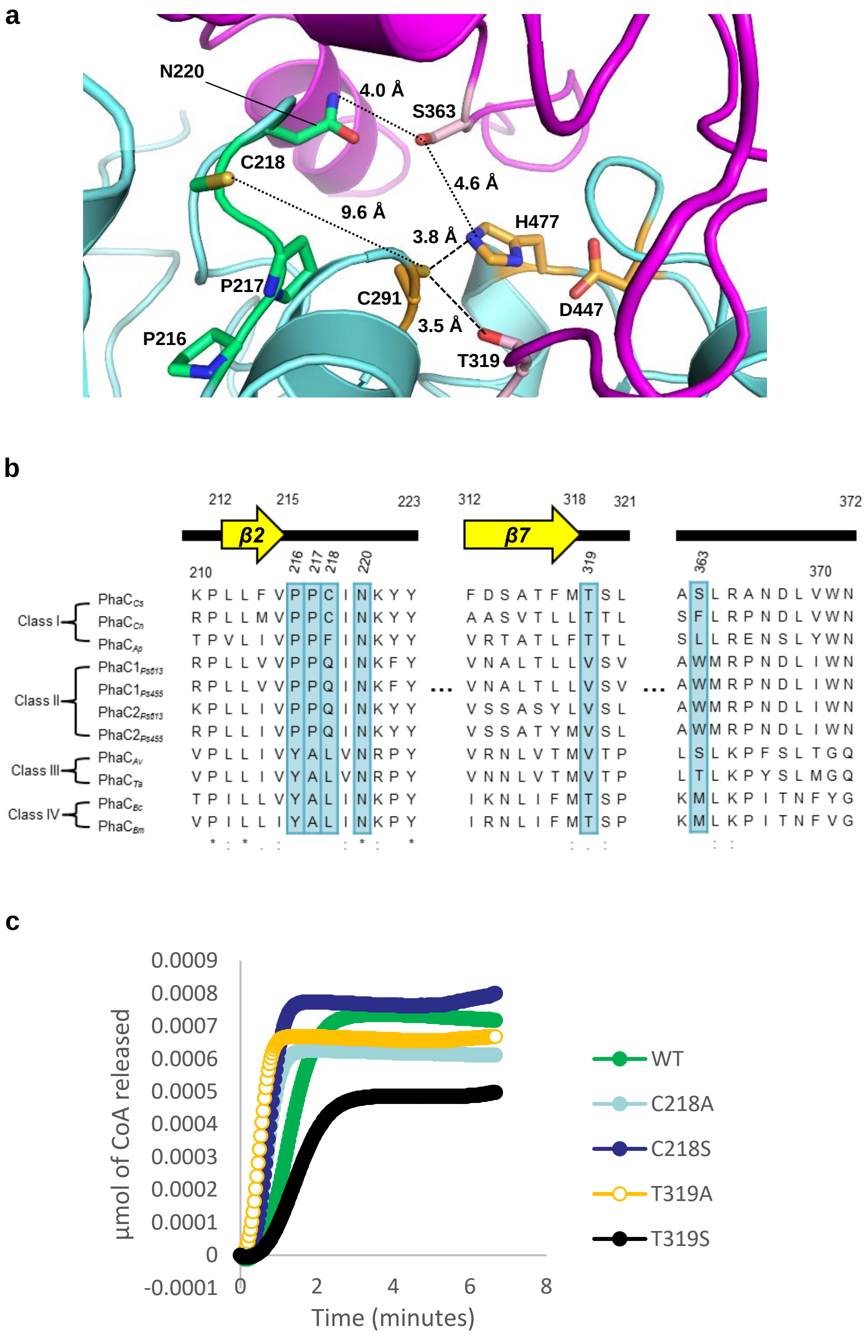

Supplementary Figure 6

The active site of PhaC$_{Cs}$-CAT contains residues at positions where assistance of active center Cys291 or intermediate binding may be possible at the active site.

(**a**) The active site of PhaC$_{Cs}$-CAT contains two polar residues, and Cys218, which may act to stabilize intermediate binding, and Thr319 may assist with activation of active center Cys291. The active site of the closed form of PhaC$_{Cs}$-CAT also contains Ser363 from the LID region of the CAP subdomain. Hydrogen bonds (broken lines) and interesting distances (dotted lines) are indicated.
(**b**) Partial alignment of the sequence showing Cys218, Thr319 and Ser363 of PhaC$_{Cs}$-CAT.
(**c**) Enzymatic activity of wild-type and mutant PhaC$_{Cs}$ with substrate 3HB-CoA. Released CoA was monitored.

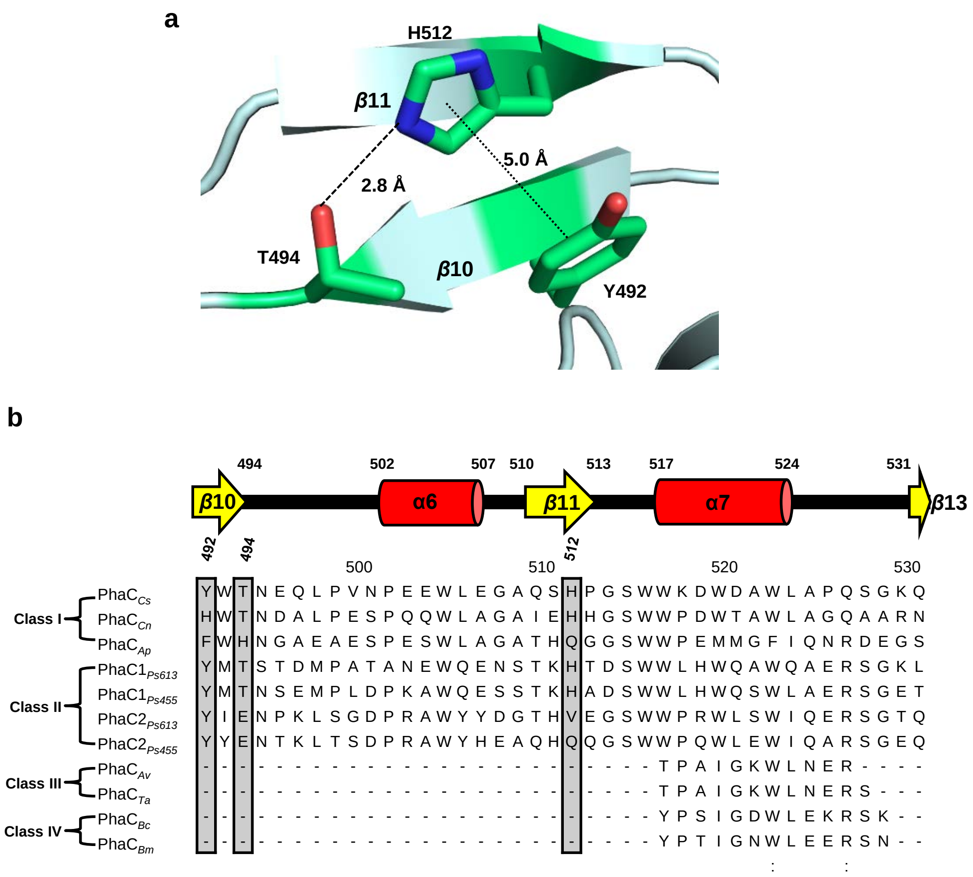

Supplementary Figure 7
Conformation of the β10-β11 segment of PhaC$_{Cs}$.

(**a**) A close-up view of the β9-β10 antiparallel β-sheet, which is part of the PhaC-specific additional β9-α5-β10 segment of the core subdomain. The segment is located at the edge of the α/β core and projects from the core subdomain without making contacts with the other protomer of the closed form dimer of PhaC$_{Cs}$-CAT. Hydrogen bonds (broken lines) and the ring-ring distance (dotted line) between His512 and Tyr492 residues are indicated.
(**b**) Partial alignment of the sequence showing that Tyr492 is replaced with other aromatic residues such as Phe or His in Class I synthases, but is conserved in Class II synthases.

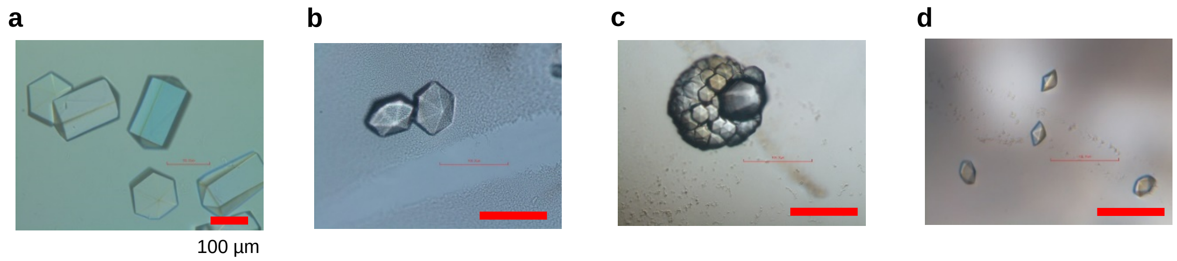

Supplementary Figure 8
Obtained crystals of PhaC$_{Cs}$-CAT.

(**a**) Hexagonal crystals of PhaC$_{Cs}$ (175-567) grown in 50 mM Bis-Tris (pH5.5), 0.06 M Ammonium sulfate, and 5% PEG4000 by seeding.
(**b**) Hexagonal crystals of full-length PhaC$_{Cs}$ grown after 8 – 12 months in a sitting drop set up equilibrated against INDEX (75): 0.1 M Bis-Tris (pH6.5), 0.2 M Lithium sulfate, and 25% PEG3350.
(**c**) Crystal clusters of α-chymotrypsin-digested PhaC$_{Cs}$ in a hanging drop against 0.1 M Bis-Tris (pH6.5), 0.2 M Lithium sulfate, and 20% PEG4000.
(**d**) α-chymotrypsin-digested Se-Met labeled PhaC$_{Cs}$ grown in 0.1 M Bis-Tris (pH6.3), 0.2 M Lithium sulfate, and 15% PEG3350 by seeding.
